# Phonetic compliance: a proof-of-concept study

**DOI:** 10.3389/fpsyg.2014.01375

**Published:** 2014-12-04

**Authors:** Véronique Delvaux, Kathy Huet, Myriam Piccaluga, Bernard Harmegnies

**Affiliations:** ^1^Laboratoire de Phonétique, Institut de Recherche en Sciences et Technologies du Langage, Université de MonsMons, Belgium; ^2^Fonds National de la Recherche ScientifiqueBrussels, Belgium

**Keywords:** phonetic compliance, L2 learning, individual skills, foreign language aptitude, psychometric test, phonetic talent, phonetics and phonology

## Abstract

In this paper, we introduce the concept of “phonetic compliance,” which is defined as the intrinsic individual ability to produce speech sounds that are unusual in the native language, and constitutes a part of the ability to acquire L2 phonetics and phonology. We argue that phonetic compliance represents a systematic source of variance that needs to be accounted for if one wants to improve the control over the independent variables manipulated in SLA experimental studies. We then present the results of a two-fold proof-of-concept study aimed at testing the feasibility of assessing phonetic compliance in terms of gradient. In study 1, a pilot data collection paradigm is implemented on an occasional sample of 10 native French speakers engaged in two reproduction tasks involving respectively vowels and aspirated stops, and data are analyzed using descriptive statistics. In study 2, complementary data including L1-typical realizations are collected, resulting in the development of a first set of indicators that may be useful to appropriately assess, and further refine the concept of, phonetic compliance. Based on a critical analysis of the contributions and limitations of the proof-of-concept study, general discussion formulates the guidelines for the following stages of development of a reliable and valid test of phonetic compliance.

## Introduction

This paper reports on a proof-of-concept study aimed at testing the feasibility of assessing “phonetic compliance,” i.e., the intrinsic speaker-specific ability to appropriately mobilize speech perception and production processes in order to produce, in a controlled way, speech sounds that are unusual in his/her mother tongue.

In a first section, we argue that a significant part of the inter-individual variation commonly reported in experimental studies on L2 sound learning is in fact due to an unaccounted-for systematic source of variance which is related with individual phonetic skills, and deserves to be independently considered. We then define the concept of “phonetic compliance,” in the context of the existing literature on general “foreign language aptitude” and specific ability to process nonnative speech sounds, including “phonetic talent.”

Second, we report the results of a two-fold proof-of-concept study in which we tested the feasibility of assessing phonetic compliance on a sample of 10 French speakers engaged in the as-faithful-as-possible reproduction of unfamiliar vowels (here, oral, static vowels) and consonants (here, long VOT syllable-initial stops). As is often the case in the development of psychometric tests, we show how this first implementation study leads both the concept itself and the methods used to measure it to refine and build off each other.

Third, in the general discussion, we evaluate the contributions and limitations of this feasibility study in the perspective of follow-up, larger scale, studies aiming at assessing phonetic compliance in French speakers, then we outline the major requirements for building a reliable and valid test of phonetic compliance that would be applicable to speakers from any linguistic background.

### Toward a new paradigm for research on L2 speech sounds processing

In previous studies focusing on how speakers process nonnative speech sounds, large amounts of inter-individual variability have often be reported (Piske et al., 2001; Francis and Nusbaum, 2002; Moyer, 2004; Golestani and Zatorre, 2009; Jilka, 2009; Flege and MacKay, 2011). Specifically, in experimental studies investigating the effects of independent variables on the subjects' performances (e.g., control for linguistic background and individual history with foreign languages, manipulations of the sounds to imitate, use of contrasted training processes, etc.), inter-individual variability often turns out to be so strong that it dramatically hampers the assessment of the effects of the independent variables themselves (Piccaluga et al., 2011; Delvaux et al., 2013).

Traditionally, in human sciences (and especially in behavioral sciences), inter-individual variation in groups is considered as a random effect resulting from the sampling process, i.e., as measurement *noise*. At the individual level, the classical model of the True Score Theory (TST) takes into account a noise component, since it claims that any human behavior measurement can be expressed as:

(1) X=T+E

where *X* is an actual observed score and *E* is the measurement error. In that framework, *E*, the error, is viewed as a *random* component resulting from the combined action of all the non-controlled sources of variance, provided that they are all weak and of equal importance (i.e., none of them is substantially greater than the others). *T* is the *true score*, i.e., the score that would have been actually observed in the absence of measurement error. In other words:

(2) E→0⇒X→T

Both *T* and *E* are unobservable theoretical constructs, whereas *X* is the mathematical expression of an observed behavior. Under the assumptions of the TST, for any given human subject and any measurement device, *T* is a steady value characterizing the conjunction of this specific subject with this specific device (Allen and Yen, 1979). All along the Twentieth century, the major techniques of psychological testing, as well as important models of statistical data treatment (e.g., factorial analysis and analysis of variance), have been based on similar conceptions.

In experimental devices studying L2 sound learning under the effect of independent variables, performances' assessment can be modeled in the same view, so that T expresses the result of the engagement of a given speaker in a given phonetic task. The experimental task involves a series of systematic sources of variance (the controlled variables *v*_1_ *to v*_*n*_ in Equation 3), and T variability can be viewed as the result of their concurrent actions, i.e.:

(3) $$\sigma_{T}^{2}\;=\sigma_{V1}^{2}\;+\sigma_{V2}^{2}+\;\ldots +\;\sigma_{Vn}^{2}\;+\;\sigma_{interactions}^{2}$$

Actual measurements should therefore be considered as depending, on the one hand, upon the variability of the systematic sources of variance, and on the other hand upon the variability of the error:

(4) $$\sigma_{X}^{2}\;=\sigma_{V1}^{2}+\sigma_{V2}^{2}+\;\ldots +\sigma_{Vn}^{2}+\sigma_{interactions}^{2}+\sigma_{E}^{2}$$

From the experimenter's viewpoint, it is very important to minimize σ^2^_*E*_ in order to maximize the weight of σ^2^_*T*_in σ^2^_*X*_, (i.e., to improve the reliability), so as to allow the most accurate observation of the independent variables' effects. In the field of psychometrics, this is usually achieved by a finely-tuned control over experimental procedures and, more generally, by paying careful attention to methodological issues. Yet, in most studies involving the production of L2 speech sounds, high variances of the X scores remain, although careful precaution has been taken in controlling the variables in the paradigm.

Since σ^2^_*X*_ magnitude is unlikely caused by random processes *only*, another, independent source of variance has to be taken into account. As it is not “error” in the TST sense, it must be viewed as systematic, and therefore as part of the determinants of T. This new component (hereafter, referred to as the C component) should nevertheless be distinguished from the controlled sources of variance of the experimental paradigm, because it is not under the experimenter's control. Equation 4 should then be re-written:

(5) $$\sigma_{X}^{2}=\sigma_{V1}^{2}+\sigma_{V2}^{2}+\ldots +\sigma_{Vn}^{2}+\sigma_{C}^{2}+\sigma_{interactions}^{2}+\sigma_{E}^{2}$$

In other words, the C component has initially been misinterpreted as part of the random error E, whereas it is in fact a systematic, non-random factor, independent from E.

In our view, the C component expresses what we call the subject's *phonetic compliance*, i.e., his/her individual ability to produce unfamiliar speech sounds he/she is exposed to, and therefore his/her ability to mobilize speech perception and production processes in order to cope with the requirements of the task.

Indeed, from a critical analysis of the literature (detailed below), it is sensible to consider that: (i) speakers differ from each other in terms of general phonetic ability, so that they differ in their performances in a variety of experimental tasks involving unfamiliar speech sounds processing; and that: (ii) this general ability is an intrinsic, rather stable characteristic of any individual, at least over a given period of time. This is, for instance, in line with the experience of second language acquisition (SLA) teachers, who deal on a daily basis with classmates exhibiting a wide range of abilities to produce and perceive nonnative speech sounds (Ellis, 2004; Mangubhai, 2006). Phonetic compliance, the intrinsic ability of adult speakers to produce, in a controlled way, unfamiliar speech sounds they are faced with, notwithstanding the differences between these sounds and the sounds they are used to process, is the focus of this paper.

Of course, the model in Equation 5 only deserves interest if (i) evidence can be found that phonetic compliance does actually vary among individuals but is relatively stable within each individual, and if (ii) phonetic compliance can practically be assessed in terms of gradient. Then, procedures for the assessment of phonetic compliance could be developed, which would allow controlling for this factor in experiments focusing on the production of L2 speech sounds. Indeed, if C can be empirically measured and therefore does no longer remain a theoretical construct, its effect could be removed from the empirically observed X variability, thus delivering better estimates of the independent variables' effects, so that:

(6) $$\left(\sigma_{X}^{2}-\sigma_{C}^{2}\right)-\sigma_{T}^{2}<\left(\sigma_{X}^{2}-\sigma_{T}^{2}\right)$$

To sum up, we posit that the large inter-individual variability which is usually observed in experimental studies on L2 sound learning actually derives from two sources of variance: on the one hand, the variability traditionally attributed to the nature of the subjects' selection process (E in the TST sense), and on the other hand, the systematic variability related with phonetic compliance, an ability which is inherent to each subject but substantially varies from subject to subject (part of the determinants of T). We claim that, in order to appropriately assess the performances of speakers who are engaged in a L2 sound learning task, one should both reduce the influence of E in X, so as to improve reliability, and remove the effects of C from X, so as to improve validity. The latter requires an independent assessment of phonetic compliance.

In order to empirically measure phonetic compliance, it is necessary to build up an appropriate psychometric test characterized by (i) specific tasks aimed at eliciting observable behaviors revealing as uniquely as possible the subject's phonetic compliance; and (ii) mathematical tools enabling to isolate the most relevant aspects of the observed behaviors and to express them in numeric form. As a first step, a pilot device should be developed and tested for reliability, before entering the classical iterative process of building up the construct validity.

In the next section, we further define the concept of phonetic compliance, showing how it fits in the context of SLA studies, then we specify the goals of the present proof-of-concept study designed at testing the feasibility of phonetic compliance assessment.

### Background

#### General foreign language aptitude

General foreign language aptitude has been a long-standing issue in SLA literature. Pioneer work was carried out by Carroll and colleagues (Carroll and Sapon, 1959; Carroll, 1981). Foreign language aptitude was considered as a relatively immutable (innate, fixed, and invariable in L2 development) specific ability for language learning, that is separated from general intelligence and motivation. In Carroll's view, this ability does not account for the failure or success of learners but it can justify why some learners learn foreign languages more quickly than others (Carroll, 1981). Carroll proposed a model of ability including four components (phonemic coding ability, grammatical sensitivity, inductive language learning ability, and associative memory), and developed the related Modern Language Aptitude Test (MLAT: Carroll and Sapon, 1959; latest version: Carroll and Sapon, 2002). Pimsleur (1966) developed an alternative test (The Pimsleur Language Aptitude Battery; PLAB) that placed more emphasis on auditory factors and less on memory (Dörnyei and Skehan, 2003). These two tests are the ones most commonly used in aptitude research, even if they have been largely criticized over the years because they do not encompass the full range of learner-, task-, and context-related factors that are likely to exert influence on the measurements (Parry and Stansfield, 1990).

Indeed, these early works were strongly linked with the—prevailing at that time—views of language, language learning, and language teaching, respectively as structuralist, behaviorist, and audio-lingual (Ellis, 2004). As these views were challenged, so interest in aptitude declined (Khatib et al., 2010). However, a renewal of interest took place over the last decade, first within the framework of information processing theory (Dörnyei and Skehan, 2003), and second through the development of the interactionist model of aptitude complexes (Robinson, 2001, 2005, 2012). In the latter model, a componential framework is proposed for mapping the interactions between tasks demands, context properties and learner factors. Aptitude is not considered to be one-dimensional in nature; it rather has a hierarchical but multilevel nature which is referred to as an “aptitude complex.”

#### Specific ability for processing nonnative speech sounds

The ability for acquiring L2 phonetics and phonology is usually considered as a rather independent subcomponent of the overall language ability (e.g., Schneiderman and Desmarais, 1988), so that there is a potential dissociation between poor speech production performances and a good mastering of L2 syntax and vocabulary (the so-called *Joseph Conrad phenomenon*: Scovel, 1988). The *Phonemic Coding Ability* (Carroll, 1981) is defined as an ability to identify new language sounds or strings of sounds and to store them in long-term memory. More recent models of phonological working memory as a language learning device (Baddeley et al., 1998; Baddeley, 2003) also focus on the perceptual side of the ability for acquiring L2 phonological systems.

Recently, Jilka and collaborators proposed a comprehensive approach for testing “phonetic talent,” which assesses phonetic abilities of adult experienced L2 learners with a special emphasis on pronunciation (Jilka et al., 2007; Jilka, 2009). In this approach, individual phonetic abilities are evaluated through a variety of speech production and speech perception tasks in L1 (German), L2 (English), and a non-familiar language (Hindi), and complementary psychological and personality characteristics are documented through a comprehensive battery of questionnaires. Data analysis leads the authors to classify learners into two groups, the high-aptitude and the low-aptitude learners, to be selected for further neuroimaging experiments. As stated by the authors, their approach does not provide a way of clearly separating between “phonetic talent” and other variables potentially driving the performances, such as L2 proficiency (and, to a minor extent, motivation). In the absence of an “experimental method that directly assesses exclusively phonetic talent,” they choose to approximate the notion “via the combination of many different tests” (Jilka, 2009, p. 41). One consequence of this approach is that it results in an exceptionally large amount of data. In order to reduce the multidimensionality of the production data, the performances in production undergo no acoustic analysis, but are assessed through subjective perceptual judgments, either by native raters or by experts referring to an expected model, resulting in an evaluation of the participants' “accent” to be compared with other (psychological, cognitive, and linguistic) measures in a large correlational analysis. Though interesting because of the wide span of its analysis, this research provides no direct measurement of the speakers' performances in the speech production domain.

To go more deeply in the analysis of the work undertaken by Jilka and collaborators, it has to be emphasized that the notion of “phonetic talent” itself is not suited to our concern. Indeed, in the framework of language aptitude described above, phonetic talent denotes a largely *innate*, neurobiologically grounded, individual skill, which is part of general language aptitude (but is not related to other specific linguistic skills such as grammatical talent in L2). Trying to assess phonetic talent would therefore necessitate distinguishing between this initial predisposition and the other interacting variables that have contributed to each individual's language development and may still influence his/her productions in a specific task, independently of his/her proficiency in any L2. In the terminology adopted by Gagné (2003), it would thus require distinguishing “gift” (Jilka and collaborators' “talent”), an untrained and spontaneously expressed superior natural ability, from actual “talent,” which progressively emerges from the transformation of this high aptitude into a well-trained and systematically developed skill and may benefit from the effect of a variety of other factors (Gagné, 2003).

### Research goals

Our approach is quite less ambitious but more direct and pragmatic, in that we purposely adopt a task-oriented view, with no strong hypothesis about the complex etiology of the phenomena we observe. Our focus is the “here and now” speaker-specific phonetic ability to produce (and indirectly, to perceive) nonnative speech sounds *as it is revealed by* his/her actual behavior in specific tasks involving the production of speech sounds that are unusual in his/her native language. In that sense, the notion of phonetic compliance encompasses both: (i) the competence that allows a controlled production of unfamiliar speech sounds, and (ii) the performances resulting from the actualization of that competence in specific paradigms.

When restricted to (i), phonetic compliance may be viewed as part of the component of the overall foreign language aptitude that deals with nonnative speech sounds. We consider phonetic compliance as one of the abilities that a foreign language learner relies on when acquiring L2 phonetics and phonology. In other words, being sufficiently phonetically compliant is a necessary, but not sufficient, condition to master the phonetics and phonology of a foreign language. Other phonetic/phonological skills, such as the ability to build from extensive exposure to a given foreign language the appropriate mental representations (and associated behavior) for language-specific coarticulatory patterns, allophonic variation and phonetic categories (including magnet effects and prototypes), are beyond the scope of phonetic compliance.

Besides, phonetic compliance is not a fixed innate ability, but the ever-evolving skill that emerges from the interaction between speaker-specific linguistic, cognitive and psychological determinants and individual experience including training in specific foreign languages. In line with most recent views on aptitude complexes, when assessing phonetic compliance the resulting performances represent the interactions between tasks demands, context properties and learner factors.

To our knowledge, there is currently no report in the literature of a preceding attempt to study phonetic compliance as itself, independently of proficiency in any specific L2, and based on direct measurements on speech production data.

The distant aim of this line of research is to provide the community with a reliable psychometric test of phonetic compliance. Such a test would allow investigating phonetic compliance in itself as well as its relationship with other variables, e.g., how it is related to intrinsic individual variables such as motivation, general linguistic abilities, short-term memory abilities, individual strategies in L2 learning, propensity to phonetic convergence in L1, etc. In addition to a better understanding of the behaviors of individuals engaged in language learning, we believe that SLA experimental research may benefit from tools allowing to control for phonetic compliance, e.g., by constitution of balanced experimental and control groups or by the use of statistical compensatory processes such as covariance analysis.

In this paper, we present a two-fold proof-of-concept study which constitutes the first stage of a research program aimed at the development of phonetic compliance assessment tools. First, we propose a pilot data collection paradigm, which is based on a reproduction task involving different speech materials, and we explore how an occasional sample of subjects copes with it, using a variety of statistical analyses (study 1). Second, we develop three customized indices, each aimed at providing a first numeric counterpart of the participants' phonetic compliance in its specific way (study 2). Note that, in accordance with a proof-of-concept approach, the selected speech materials and group of participants are not intended to represent the full range of behaviors likely to arise from the target population in relation with phonetic compliance. Rather, they have been selected as part of an adequate laboratory setting to instantiate the phenomenon under study. Likewise, the set of indicators that is drawn from this pilot data collection paradigm is not intended to be final, but will be used as a basis of discussion for further improvements.

## Study 1

### Materials and methods

#### Stimuli

Two stimuli sets, a “vowel set” and a “VOT set” have been built using Klatt's synthesizer ^1^ in order to ensure a total control of the acoustic parameters determining the resulting acoustic signals (Klatt, 1980).

**Figure 1:**
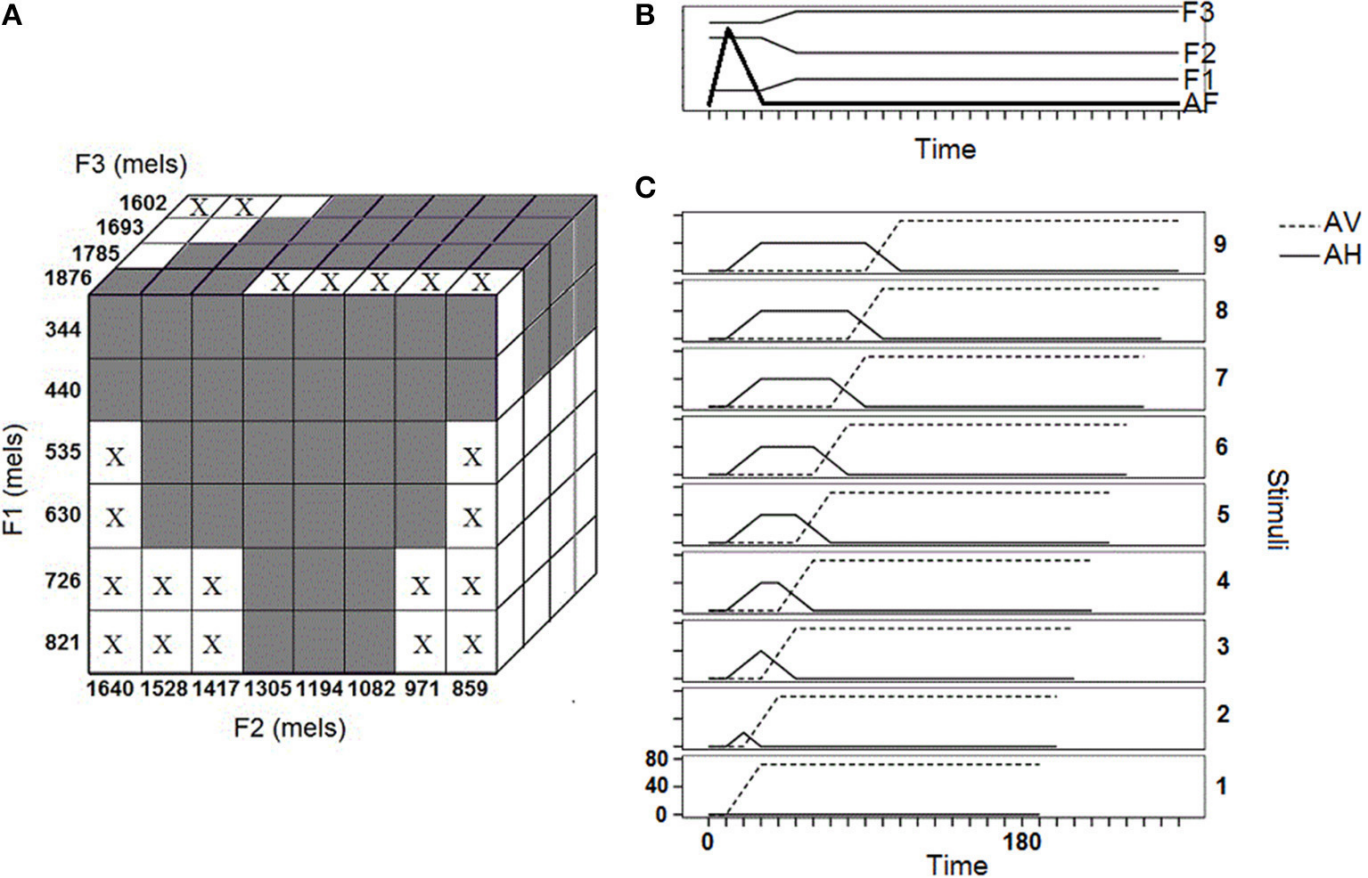
**Stimuli properties: (A) Vowel stimuli properties in the F1∗F2∗F3 space; (B) Time evolution of the Klatt's synthesizer parameters taking similar values over the 9 VOT stimuli; (C) Time evolution of the Klatt's synthesizer parameters varying across the 9 VOT stimuli**.

The vowel set is made of 94 synthesized vowels that are evenly distributed over a mel scale F1∗F2∗F3 acoustic space (Figure 1A). Total vowel duration (200 ms) and F0 contour (from 110 to 90 Hz) are kept constant across stimuli. The first three formant values vary across stimuli, whereas F4 and F5 are kept constant and virtually annihilated by applying to them a fixed bandwidth value of 1000 Hz.

F1 varies from 344 to 821 mels by steps of 95.4 mels, F2 varies from 859 to 1640 mels by steps of 111.5 mels; F3 varies from 1602 to 1876 mels by steps of 92.3 mels. Formant bandwidths consist in 70% of the critical band (Zwicker and Fastl, 1999) at the corresponding frequency. Formant frequencies boundaries have been fixed in relation with the documented formant properties of the vowels of the world's languages (Ladefoged, 2005, p. 175). The maximal number of steps in each dimension (respectively: 6 along F1, 8 along F2, and 4 along F3) has been set so that it exceeds the number of possible phonemic distinctions in the world's languages (IPA, 1999), but not so much so that the total number of stimuli would be impracticable. Finally, some combinations of F1/F2/F3 values have been excluded because they do not exist in natural languages due to the limitations of the articulatory-acoustic space imposed by the properties of human vocal tract (crosses in Figure 1A).^2^

The VOT stimuli set is made of 9 [ka] synthetic syllables, where [k] varies in voice onset time (VOT) from virtually nil VOT [k] to long positive VOT [k^h^]. The [a] vowel is the same across stimuli, with duration of 160 ms and an appropriate descending F0 contour (from 110 to 90 Hz).

Across the 9 stimuli, the time courses of Amplitude of Frication noise (AF) and of F1, F2, F3 frequencies are similar (Figure 1B). A burst of 20 ms is achieved by increasing AF from 0 to its maximal value in the first 10 ms of the sound signal, then decreasing it back to 0 in the following 20 ms. At time 30 ms (t_30_), stop released is achieved and 20-ms formant transitions appropriate for a velar-to-[a] transition start. From t_50_ to the end of the sound signal F1, F2, F3 are set at respectively 700, 1400, and 2500 Hz.

The 9 stimuli vary in the time evolution of two parameters, namely Amplitude of aspiration noise (AH), and Amplitude of Voicing (AV) (Figure 1C). Overall, from stimulus 1 to stimulus 9, voicing is set off later and later with respect to stop release (t_30_), whereas the aspiration phase lasts longer and longer, which results in ever more positive VOT. For a given stimulus, AH, and AV turning points are synchronous, so that AV starts its increase when AH starts its decrease and AV reaches its maximum when AH is back to zero (Figure 1C). Acoustically, stimuli 1–9 vary from a 20 to a 100 ms VOT.

#### Participants

The participants are 10 native French speakers from Belgium, 5 female (S1 to S5), 5 male (S6 to S10), aged 24–42, who have all completed a 3- or 5- years degree in a higher education institution. They were administered a comprehensive “linguistic questionnaire” in which they detailed their knowledge and experience with foreign languages. Table 1 reports the participants' self-rating scores (on a 10-point scale) in oral comprehension and oral production for any relevant foreign language. In the table, “school” refers to participants who rated their oral comprehension and production as inexistent (zero on the 10-point scale) although they've had some experience of the language through L2 classes in high school.

**Table 1:** **Participants in study 1: age, gender, and self-rating scores (on a 10-point scale) in oral comprehension-oral production for any relevant foreign language**.

**Speaker**	**Age**	**Gender**	**English**	**Dutch**	**Other**
1	24	Female	8–6	School	Italian 9–7
2	25	Female	8–6	School	None
3	42	Female	7–6	School	None
4	30	Female	3–3	School	None
5	32	Female	9–8	5–3	Spanish 9–7
6	33	Male	8–6	School	German 1–1
7	28	Male	8–6	None	Spanish 2–1
8	28	Male	6–5	3–2	None
9	28	Male	7–5	8–7	Spanish 3–1
10	31	Male	9–9	9–9	None

Considered together, the reported self-rating scores can be considered as typical of well-educated young adults born and living in the French-speaking part of Belgium (Ginsburgh and Weber, 2006; Blondin et al., 2008) in that: (i) English is by large the privileged foreign language; (ii) although 9 participants out of 10 have been exposed to Dutch in highscool, only a minority of them report any competence in oral comprehension or production in that language; (iii) other (infrequently) mentioned languages are limited to other european languages (Spanish, Italian, German).

#### Data collection paradigm

The participants sat in a sound-proof room in front of a computer screen displaying relevant information through a customized PowerPoint presentation. They could hear the stimuli through headsets which sound level was individually adjusted, and their speech was recorded via an omnidirectional Neumann U87i microphone. The participant controlled the slide to slide progress over the session (including the transition from one stimulus to the following one while the computer screen remained black) through a remote control unit.

The data collection paradigm comprised five successive parts administered in a single session. The first part consisted in the recording of L1 sounds to be used as control sounds in data analysis. The task was to read 15 items (the 14 French vowels plus the syllable [ka]) displayed one at a time on the screen following grapheme-to-phoneme conversion rules appropriate for French native speakers.

The second and third part of the paradigm consisted in respectively the training and the test phase related to the vowel stimuli set. The instructions were to “repeat the sound as faithfully as possible “as if it was a sound from a foreign language.” Participants were told in advance that some of the sounds they would hear could sound a bit unusual to them “as if they had been produced by a machine.” They were specifically asked not to attempt to mimick this “artificial quality,” if any, but to treat each stimulus as a sound coming from a foreign language. No participant reported discomfort with the stimuli and the related instructions. The training phase consisted in one repetition of 10 synthetic vowels that were selected from the stimuli set by the authors based on their (auditorily assessed) proximity with canonical realizations of French vowels. The test phase included three blocks of the 94 vowel stimuli presented in the same pseudo-random order in all blocks. No feedback was provided in either the training or the test phase.

The fourth and fifth parts of the data collection paradigm consisted in respectively the training and the test phase related to the VOT stimuli set. Instructions were again to “repeat the syllables as faithfully as possible “as if it was from a foreign language.” The training phase consisted in the reproduction of 10 [ka] synthetic syllables, 5 times stimulus 1.5 times stimulus 9, presented in alternation. The test phase included 5 blocks of the 9 stimuli presented in pseudo-random order. No feedback was provided.

Overall, raw data include 362 productions for each participant: 15 L1 productions, 10 training vowels, 282 test vowels, 10 training [ka] syllables, 45 test [ka] syllables, for a total of 3620 productions to be analyzed.

#### Data processing

The speech productions from the participants were segmented manually. Two labels were positioned on each production. For vowels, label 1 was set at vowel onset and label 2 at vowel offset based on an expert's visual inspection of the speech signal. For [ka] syllables, label 1 was set at burst onset and label 2 at vowel onset, i.e., at the first zero passage within the first periodic cycle of the vowel discernible on the speech signal.

For vowel productions, raw data consist in the first three formant frequencies that were first automatically detected in the middle of the vowel using Praat (with adapted parameters for female speakers), then manually verified by two trained phoneticians examining spectrograms. For [ka]-syllable productions, VOT measures consist in the duration of the interval between label 1 and label 2.

For vowel productions, the distance between the target (the stimulus) and the response was computed as the Euclidean distance between them in the three-dimensional F1∗F2∗F3 acoustic space defined in mels:

(7) $$\text{Distance}={\left[\sum_{\text{i }=\;1}^{3}{\left({\text{Fi}}_{\text{stimulus}}\text{ - }{\text{Fi}}_{\text{response}}\right)}^{2}\right]}^{1/2}$$

### Data analysis

#### Vowel stimuli

**Figure 2:**
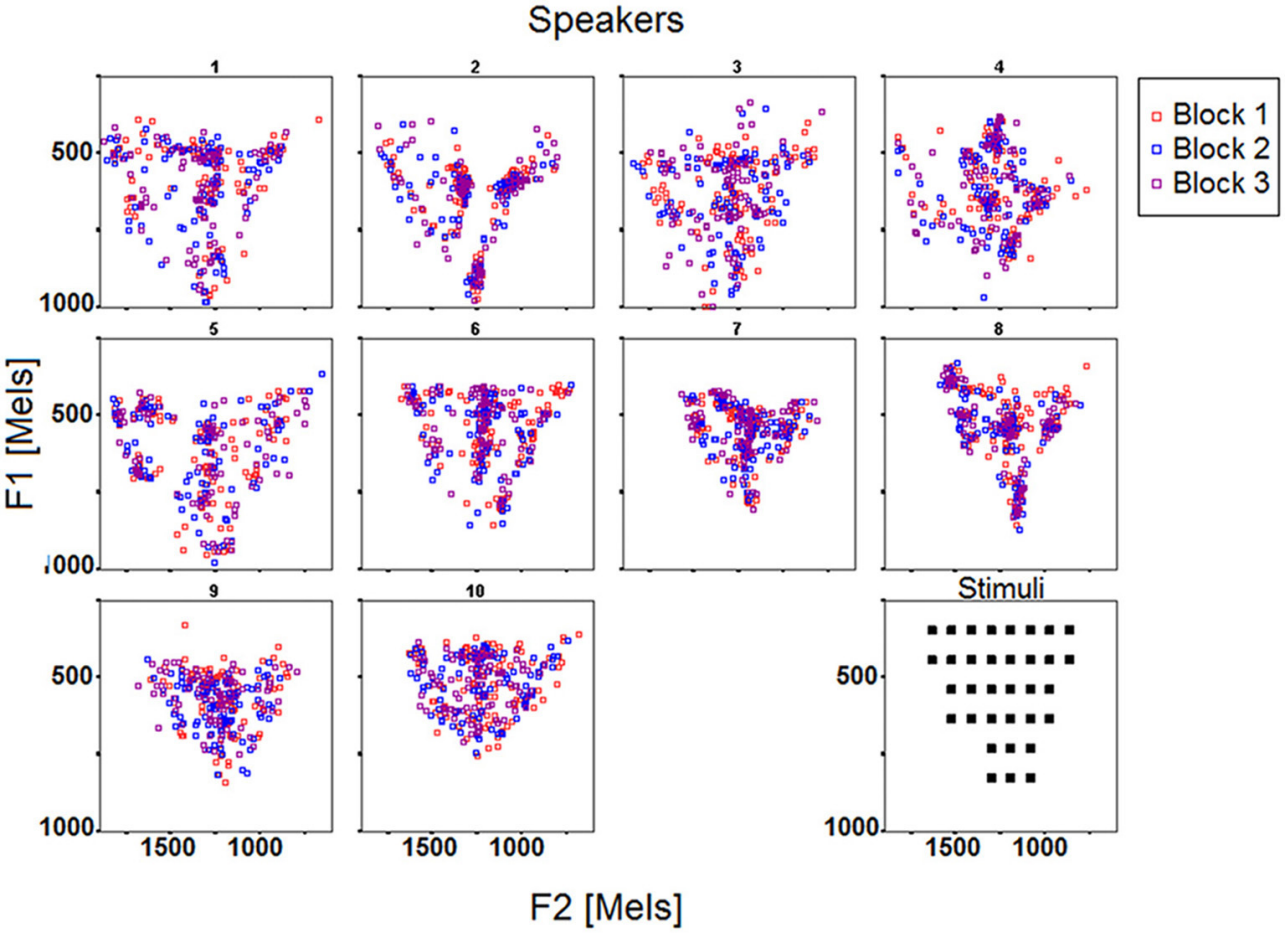
**F1 and F2 (mels) of the stimuli and response vowels across the 10 speakers and the 3 blocks**.

**Figure 3:**
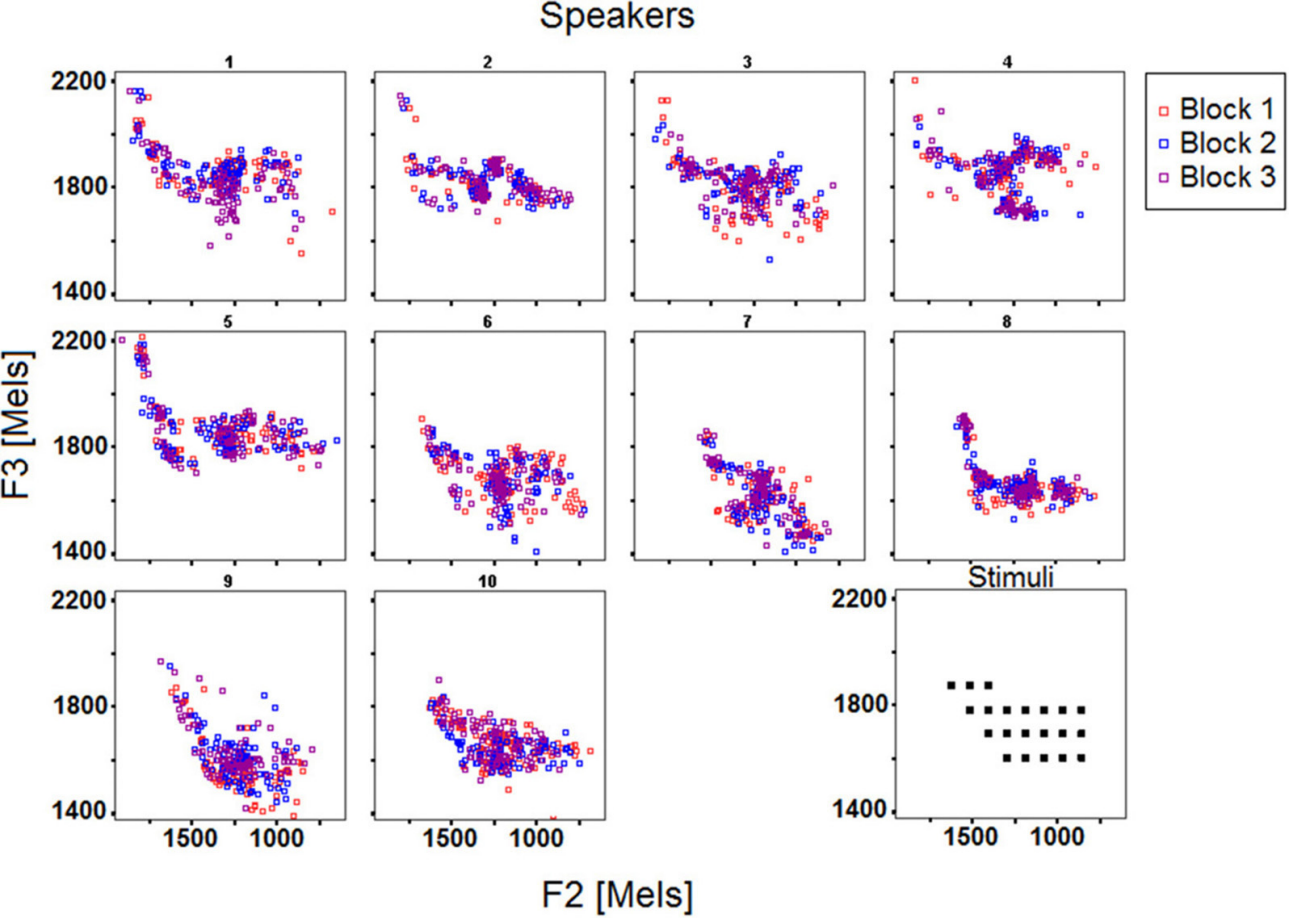
**F2 and F3 (mels) of the stimuli and response vowels across the 10 speakers and the 3 blocks**.

***Overview of the data***. Figures 2, 3 display the acoustic properties of the stimuli and of the response vowels elicited by the reproduction task across the 10 speakers and the 3 blocks, respectively in F1 by F2 and F3 by F2 planes (mel scale).

Three observations can be made based on the data reported in Figures 2, 3. First, it appears that each individual confines his/her productions to a particular acoustic perimeter, arguably due to anatomical, and maybe articulatory, idiosyncratic properties, which limits can differ from those of the stimuli space (e.g., S5, S7, S10 on Figure 2, S1 to S5 on Figure 3). Second, the participants differ in how their productions are distributed over their overall perimeter, some of them showing quite scattered productions, others exhibiting clusters in specific regions of the vowel acoustic space (e.g., S9, S10 vs. S2, S6). Third, from Figures 2, 3 it appears that the inter-block variability deserves further analysis.

To elaborate on these preliminary observations, the results of several analyses based on descriptive statistics are reported in the following sections. First, the similarity of each individual's responses over the three blocks was assessed using multiple correlations. Second, in a first attempt to assess the success of the subjects engaged in the reproduction task, the relationships between the targets and the speakers' responses were investigated using (per formant) group and individual linear regression analyses, as well as Euclidean distances in the F1∗F2∗F3 space. Third, individual differences in the distribution of the responses over the acoustic space were assessed based on surface density comparisons between stimuli and responses. Overall, the three types of analysis seek to describe how the speakers deal with the reproduction task involving vowel stimuli.

***Inter-blocks similarity***. The motivation for presenting each speaker thrice with the same stimuli set is to increase the volume of the collected data, leaving open the possibility of merging the data from the three blocks in further calculations. Thus, we test here that no significant effect can be attributed to the consecutive blocks by assessing the inter-blocks similarity of each speaker's productions using multiple correlations. Separately for each speaker and each formant, three multiple correlation coefficients were computed (using Block 1 and Block 2 data to predict Block 3 data, using Block 2 and Block 3 data to predict Block 1 data, using Block 1 and Block 3 data to predict Block 2 data), then averaged (Table 2). In all cases, the computed correlation coefficients turn out to be very highly significant (*p* < 0.00001). The η2 coefficients are also high, indicating that the values drawn from a given block can safely be predicted from the values drawn from the other blocks. For F1 and F2, the percentages of explained variance spread from 70% up to 90%. Note that inter-blocks similarity is nevertheless lower for F3, with larger inter-individual differences. Overall, these results indicate that, if necessary, it is fairly safe to pool data from the 3 blocks in further analyses. An additional confirmation will be brought by the analysis of variance performed on the Euclidean distances between the stimulus and the response (see infra, Relationships between targets and responses: distance analysis).

**Table 2:** **Results of the multiple correlation analysis carried out over the 3 blocks (correlation coefficients, *p*-values, η2 coefficients)**.

	**F1**			**F2**			**F3**		
**Speaker**	**r_BP_**	***p***	**η^2^**	**r_BP_**	***p***	**η^2^**	**r_BP_**	***p***	**η^2^**
1	0.925	<0.00001	0.856	0.899	<0.00001	0.808	0.687	<0.00001	0.472
2	0.871	<0.00001	0.759	0.889	<0.00001	0.790	0.870	<0.00001	0.757
3	0.861	<0.00001	0.741	0.914	<0.00001	0.835	0.789	<0.00001	0.623
4	0.837	<0.00001	0.701	0.851	<0.00001	0.724	0.728	<0.00001	0.530
5	0.949	<0.00001	0.901	0.928	<0.00001	0.861	0.882	<0.00001	0.778
6	0.936	<0.00001	0.876	0.908	<0.00001	0.824	0.606	<0.00001	0.367
7	0.910	<0.00001	0.828	0.942	<0.00001	0.887	0.875	<0.00001	0.766
8	0.911	<0.00001	0.830	0.850	<0.00001	0.723	0.646	<0.00001	0.417
9	0.898	<0.00001	0.806	0.886	<0.00001	0.785	0.615	<0.00001	0.378
10	0.934	<0.00001	0.872	0.937	<0.00001	0.878	0.722	<0.00001	0.521

Per speaker per formant analysis.

***Relationships between targets and responses: linear regression analysis***. Moving now to the relationships between the targets and the speakers' actual productions, three linear regression analyses were carried out, respectively for F1, F2 and F3, pooling together data from all 10 speakers and 3 blocks. These analyses revealed that *as a group*, the 10 speakers achieve a fair relation between their responses and the stimuli they were asked to repeat as faithfully as possible, at least in terms of F1 [R-square = 0.75; *F*_(1, 1032)_ = 3084.84; *p* < 0.001] and F2 [R-square = 0.78; *F*_(1, 1032)_ = 3598.61; *p* < 0.001] formant values (recall that the higher the coefficient of determination R-square, the higher the contribution of the stimulus variance to the response variance).

This result in turn indicates that, at least for the 10 native French speakers under study, the vowel task is appropriate in that it is neither too difficult (overall, the speakers perform fairly), nor too easy (there is no apparent ceiling effect). Moreover, given the overall high variability, the task can potentially elicit some inter-individual variation signaling individual differences in phonetic compliance. The slopes of the regression lines are respectively of 0.72 for F1 and 0.94 for F2, i.e., the responses formant values are overall closer to those of the targets in the case of F2 (a slope of 1 reflects a perfect match), whereas in the case of F1 there is an undershoot of the responses when compared to the stimuli.

Concerning F3, response formant frequencies are poorly correlated with those of the stimuli [R-square = 0.09; *F*_(1, 1031)_ = 100.4; *p* < 0.001; Slope = 0.41]. It is unclear whether this is due to F3 being more idiosyncratic (i.e., more constrained by speaker-specific anatomical characteristics or articulatory routines) than F1 and F2, especially since F3 also exhibits a poorer intra-speaker inter-blocks similarity than F1 and F2 (see Table 2). In any case, F3 is usually considered as being partly predictable from the frequencies of F1 and F2 in a given vowel, the more so in languages that don't have distinctive front rounded (or back unrounded) vowels (Ladefoged, 2005). For each block separately, as well as for the data averaged over the 3 blocks, regression analyses were carried out to assess how response F3 values can be predicted from Response F1, Response F2 and Stimulus F3 values (all speakers pooled). They all revealed that a significant part of the variance in Response F3 values can be explained by the model, Stimulus F3 contributing far less than Response F1 and Response F2 [averaged data: R-square = 0.354; *F*_(3, 1029)_ = 188.07; *p* < 0.001; partial correlations coefficients: Response F1: 0.392; Response F2: 0.411; Stimulus F3: 0.165]. Further work is necessary to determine the weight to give to F3, respectively to F1 and F2, in refined measures of phonetic compliance.

**Figure 4:**
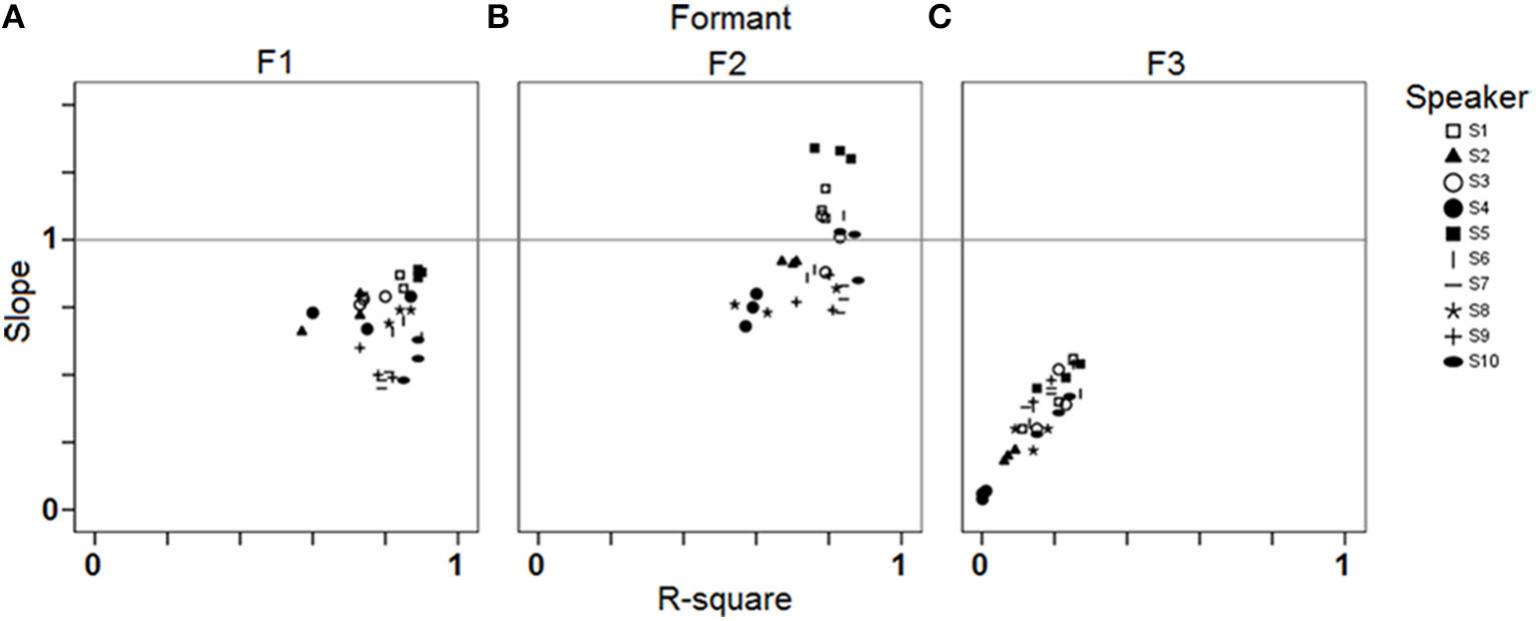
**Results of the single regression analyses carried out for each speaker and each block (study 1): slope of the linear regression line by R-square plots for (A) F1; (B) F2; (C) F3**.

In order to investigate potential *individual differences* in phonetic compliance, single regression analyses between stimuli and responses were carried out for each speaker and each block separately. Figure 4 summarizes the results of these analyses by means of slope (of the linear regression line) by R-square (coefficient of determination) plots for F1, F2, and F3. It appears from Figure 4 that these data pairs can have a fair discriminating power between individuals (especially in the case of F2), while they also allow documenting shared properties such as the overall higher slopes for the female speakers S1, S3, and S5 (Figure 4B).

***Relationships between targets and responses: distance analysis***. In order to account for the overall response-target dependency (combining information from all three formants), a distance index was computed as the Euclidean distance between the stimulus and the response in the three-dimensional F1∗F2∗F3 acoustic space (following Equation 7 above). An ANOVA was carried out with Euclidean Distance as dependent variable, Block as within-subject independent variable, and Speaker (nested in Gender) as between-subject independent variables. The statistical analysis revealed that Gender [*F*_(1, 930)_ = 267.35; *p* < 0.001], Speaker [*F*_(8, 930)_ = 5.4; *p* < 0.001], and the interaction between Block and Speaker [*F*_(8, 930)_ = 4.88; *p* < 0.001] yielded a significant variation in the computed Euclidean distances. Since the ANOVA did not reveal any significant between-Block, within-Speaker effect, overall the speakers' performances (as measured by Euclidean distances between responses and stimuli formant frequencies) did not improve or deteriorate over the short time period devoted to data collection. This provides confirmation of the evidence from Section Inter-Blocks Similarity, suggesting that the high inter-blocks formant correlations are strong enough to pool the data drawn from the 3 blocks in further computations.

**Figure 5:**
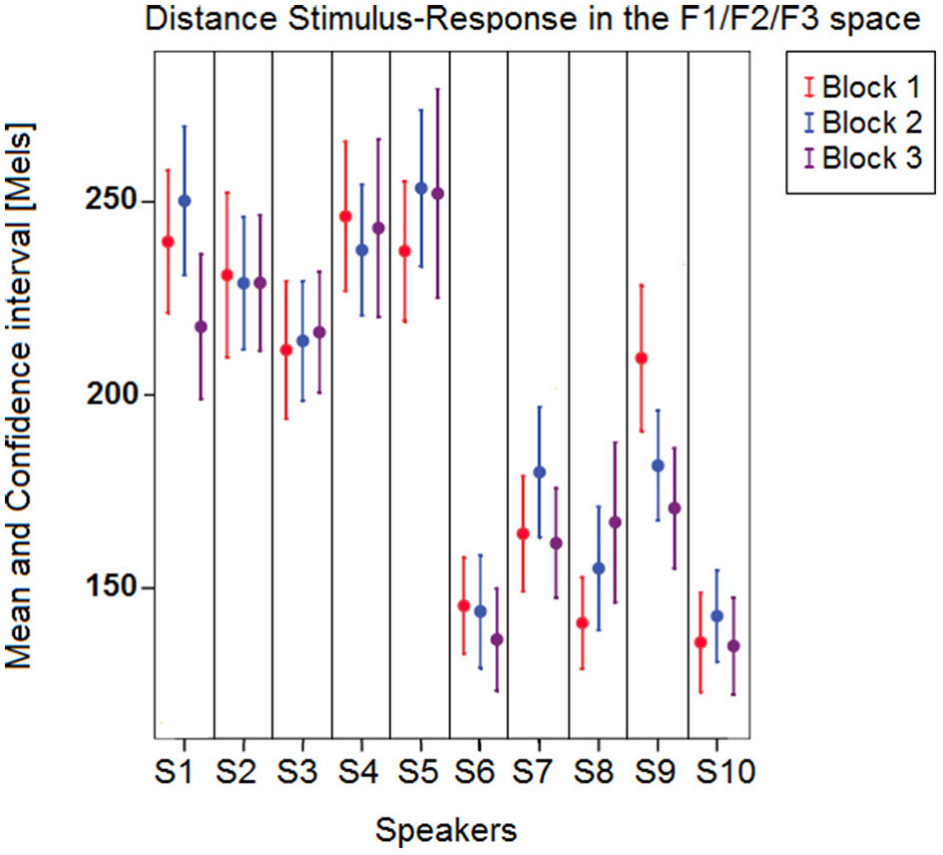
**Mean (and confidence interval) distances between vowel stimuli and responses in the F1∗F2∗F3 space across the 10 speakers and the 3 blocks**.

Figure 5 illustrates these results by plotting the mean distances as well as the corresponding confidence intervals across the 10 speakers and 3 blocks. Mean distances were larger overall for female speakers S1–S5, which was expected given that formant frequencies are generally higher for female speakers due to shorter vocal tract, and that the stimuli's specifications fell within the usual range of male voices (see the drift of the female participants' acoustic vowel space when compared to the stimuli space in Figures 2, 3). Still, the distance index allows for interindividual discriminability within gender groups, as well as it provides complementary information with respect to per-formant regression analysis (compare Figures 4, 5).

***Distribution of the responses over the acoustic space***. Individual differences in the distribution of the responses over the acoustic space were assessed by comparing the surface density of each participant's responses with that of the stimuli (in the F1/F2 plane). First, the stimuli space was partitioned in 34 rectangular zones of similar area corresponding to a projection of the stimuli's specifications on a F1/F2 plane. Then, for each speaker and each block separately, the number of responses that actually fell into each zone was compared to the number of expected responses given the properties of the stimuli using chi-squares. Proportions of observed to expected responses were also computed.

**Figure 6:**
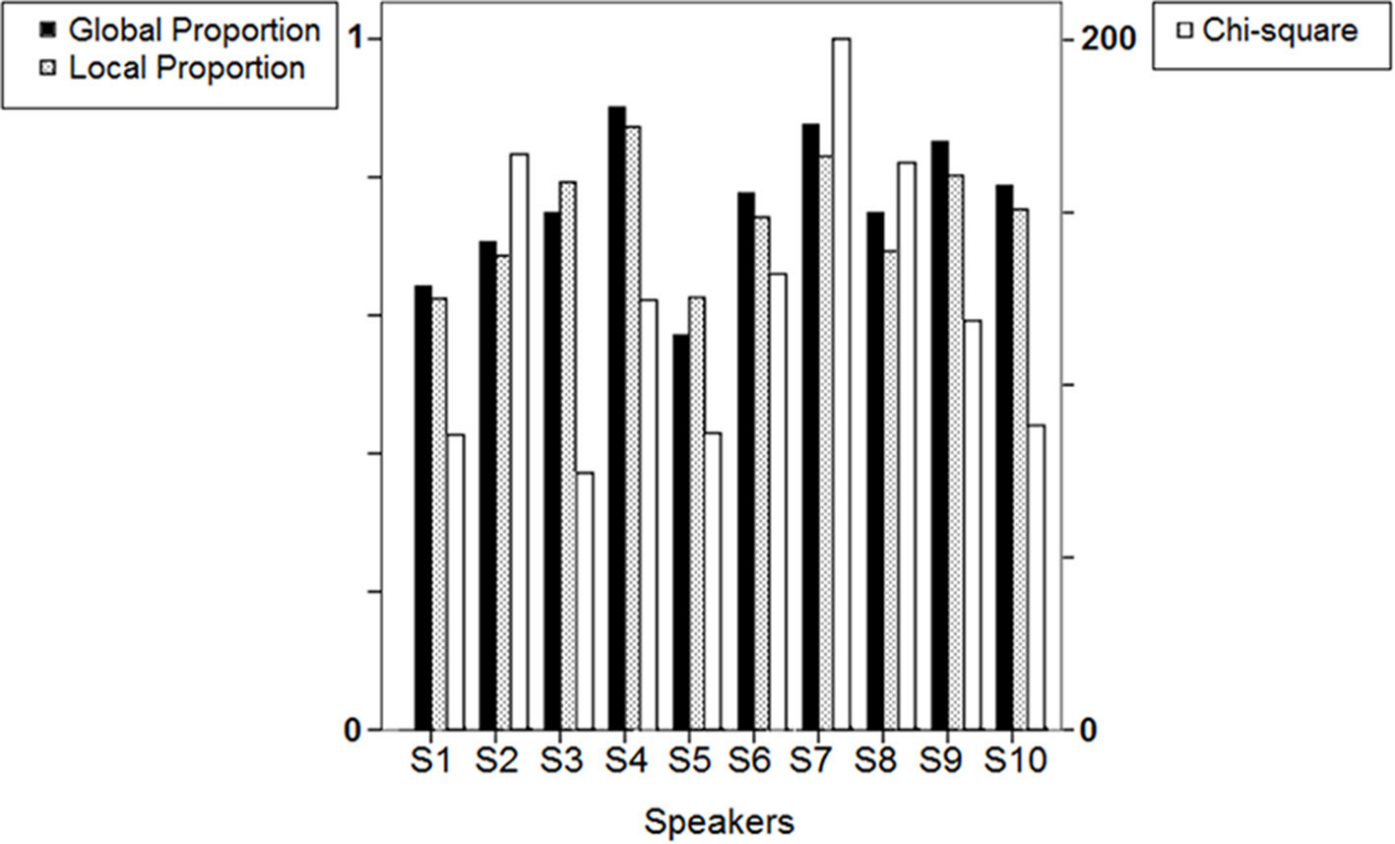
**Results of the surface density analysis: bars representing Chi-square, Local Proportion and Global Proportion means (3 blocks pooled) across the 10 speakers.** See text for details.

Figure 6 summarizes the results found for each speaker in the form of bars representing the mean values (3 blocks pooled) of three variables: (i) “Chi-square”: the sum of chi-squares for all 34 rectangular zones; (ii) “Local Proportion”: the mean proportion of observed to expected responses in each zone; (iii) “Global Proportion”: the overall proportion of observed to expected (94) responses in the supra-zone made of the 34 zones.

As illustrated in Figure 6, Local Proportion and Global Proportion yield related values for a given speaker. For 8 out of 10 speakers, the mean proportion of observed to expected responses in each particular zone is lower than the overall proportion in the supra-zone, which is consistent with the fact that the requirements are lower in the latter case for a given production to be computed as confirming the expectation.

“Chi-square” seems somewhat less related to the other two variables. S2, S7, and S8 perform the poorest according to the chi-square indicator, which reflects in part the visual impression of individual response distributions exhibited in Figure 2. Note that by examining Figure 2 one can only detect response clusters (and assume that they are close to L1 prototypes), but the display does not provide information about the relation between these responses and the corresponding targets. In other words, response clusters may include responses that are the closest-to-stimuli L1 prototypes, as well as responses that presumably correspond to L1 prototypes but largely diverge from the stimuli to be repeated.

#### VOT stimuli

**Figure 7:**
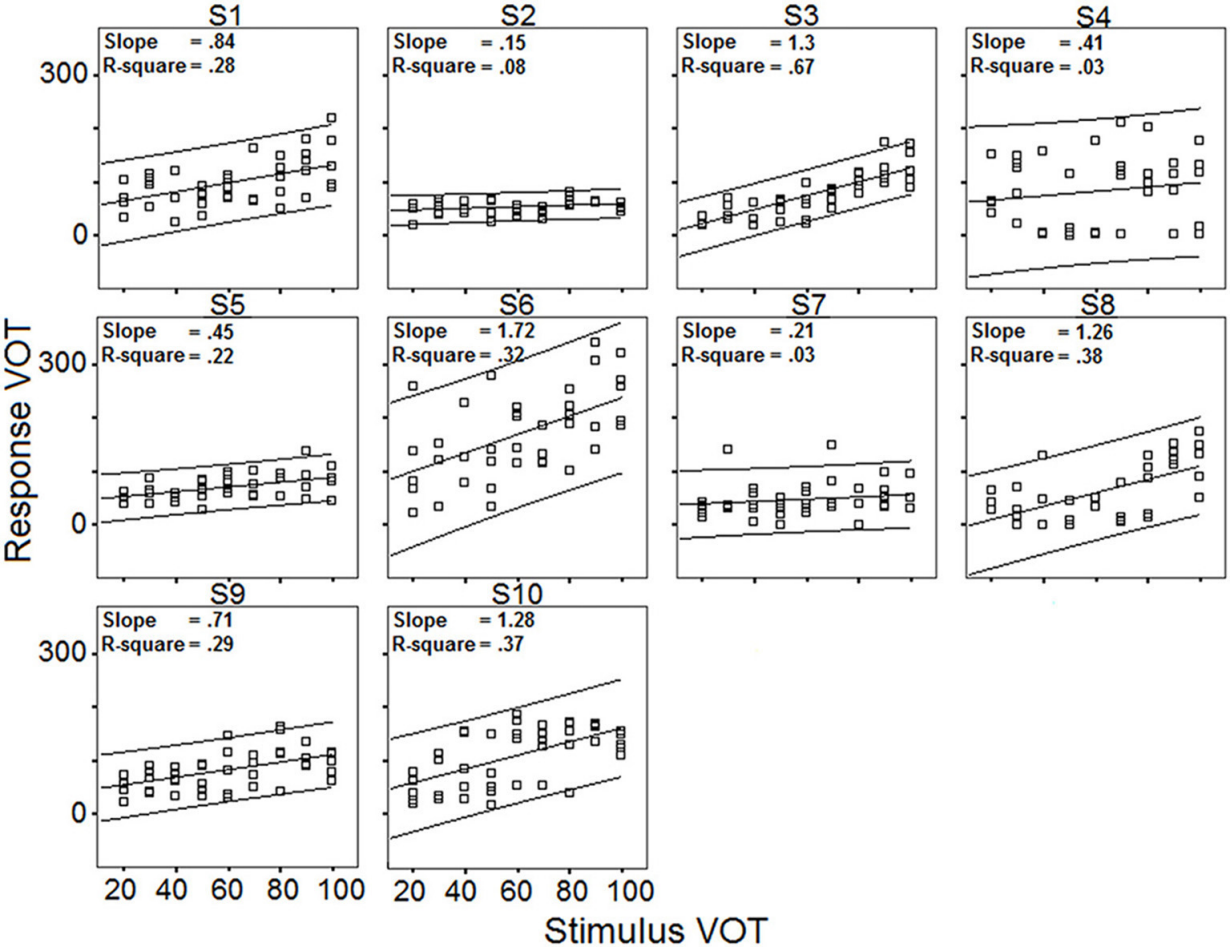
**Response VOT as a function of stimulus VOT across the 10 speakers; regression lines and 95% confidence intervals; slopes of the regression lines; coefficient of determination R-square (5 blocks pooled)**.

***Relationships between targets and responses: linear regression analysis***. Data obtained with the VOT stimuli set are shown in Figure 7, which plots response VOT as a function of stimulus VOT across the 10 speakers (5 blocks pooled). Linear regression lines (together with the corresponding slope and R-square values) with 95% individual prediction interval are also provided.

Overall, the R-square values indicate that the 10 speakers' VOT productions are moderately influenced by the stimuli VOT, or at the very least, that the speakers' productions are less influenced by the stimuli properties in the case of long VOT [k] consonants than in the case of vowels. Although the VOT measure is only one-dimensional (whereas vowel responses are assessed in a three-dimensional formant space), one should not overlook the fact that achieving long VOT for native French speakers involves acquiring new timing patterns between laryngeal and supra-laryngeal gestures.

**Figure 8:**
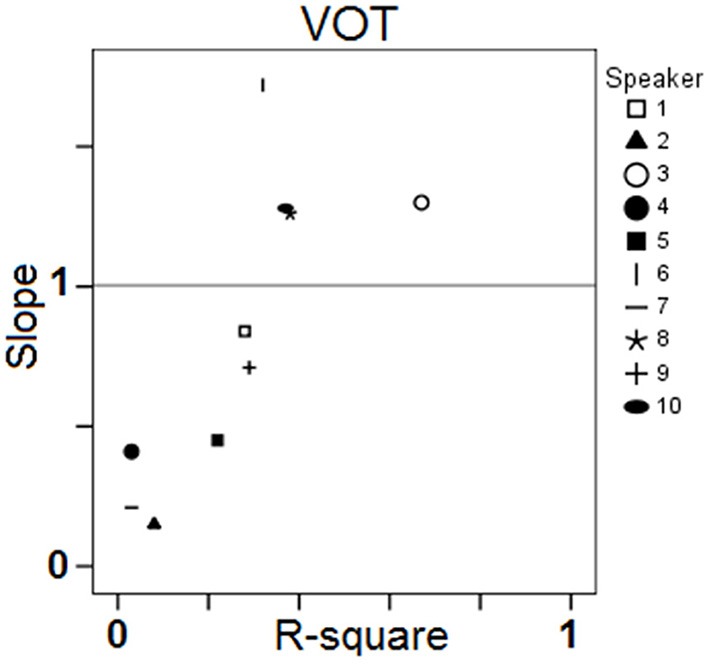
**Results of the single regression analyses carried out on VOT data for each speaker and each block: slope of the linear regression line as a function of the coefficient of determination R-square**.

Results in Figure 7 also exhibit a large amount of inter-individual variation. Some speakers achieve virtually zero R-square values with low (S2), moderate (S7), or high (S4) intra-speaker responses variability (as indicated by 95% confidence level intervals). For other speakers, there is a moderate correlation between response VOT and stimulus VOT (e.g., S1, S6, S9, S10), sometimes with large intra-speaker variability (S6). Of the 10 speakers, S3 performs the best. Figure 8 summarizes the inter-individual variation in the performances by means of a slope by R-square plot. Note that for the 6 speakers exhibiting R-square values within the range of 0.2–0.4, slopes largely vary, i.e., from.45 to 1.72.

**Figure 9:**
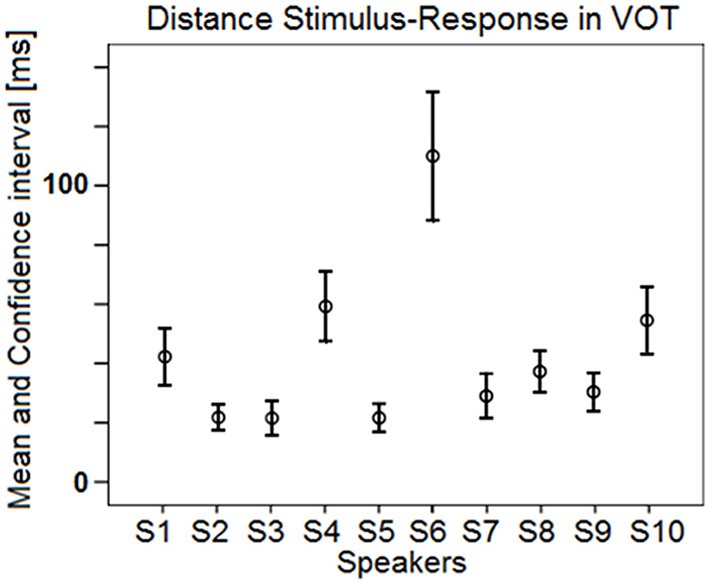
**Mean (and confidence interval) distances between stimuli and responses VOT across the 10 speakers (5 blocks pooled)**.

***Relationships between targets and responses: distance analysis***. Regarding the distance between the targets and the responses, the absolute difference, in ms, between stimulus VOT and response VOT was computed (hereafter, Distance). Then, an ANOVA was carried out with Distance as dependent variable, and Speaker and Block as independent variables. Neither Block, nor the interaction between Speaker and Block yielded significant variations in Distance, whereas Speaker did [*F*_(9, 800)_ = 21.73; *p* < 0.001]. However, this raw distance index (plotted in Figure 9) is much less efficient in terms of assessing phonetic compliance than the summary provided in Figure 8. Indeed, as shown in Figure 9, S2 and S3 achieve a very similar mean distance between response VOT and stimulus VOT (S3 having a slightly larger 95% confidence interval), i.e., respectively 21.8 and 21.5 ms, although based on the results of the regression analysis S3 performs the best whereas S2 performs poorly in the reproduction task (Figure 8). In fact, since S2 consistently produces short, L1-typical VOT in response to all stimuli (Figure 7), the mean distance from her responses to the stimuli VOT remains short when compared to that achieved by other speakers who seem to attempt to follow the stimuli, but produce some inappropriately long VOT in the process (e.g., S1, S10).

### Discussion

Overall, these first results obtained from 10 participants suggest that the selected reproduction tasks are appropriate to elicit observable behaviors relating with phonetic compliance, at least for native French speakers. From the observed range of performances, neither ceiling nor floor effect can be detected, although regression analyses comparing stimuli and responses acoustic properties indicate that the speakers generally perform better with the vowel than with the VOT stimuli. Both tasks allow for a fair range of inter-individual variation in the performances of the 10 participants as shown by the fair range of R-square and slope values achieved in the regression analyses and illustrated in Figure 4 (vowel stimuli) and Figure 8 (VOT stimuli). Using different speech materials also provides complementary information about different individual abilities. Indeed, accurately reproducing the different vowel stimuli requires that the speakers attain new, not L1-typical, targets in an acoustic/timbre space they already master, in that they are equipped with the appropriate coproduction gestural patterns. On the other hand, accurately reproducing long VOT [k] consonants requires a reorganization of the timing patterns between (already mastered) laryngeal and supra-laryngeal gestures, whereas the control of timbre is not determinant. As an example of differential behavior, S8 performs rather poorly with the vowel set, with lower than average R-square values in the regression analysis for F2 and F3 (Figure 4) and higher than average sum of chi-square values in the surface density analysis (Figure 6), whereas S8 achieves the second best performance when reproducing long VOT consonants based on the regression analysis (Figure 8).

As illustrated by the variety of data analysis procedures used above, performances in the reproduction tasks can be assessed in various ways, yielding to fairly different speakers rankings. For example, in the vowel task S5 performs better than S2 based on the regression analysis (Figure 4) whereas S2 performs better than S5 in terms of mean distance between stimuli and responses in the F1/F2/F3 space (Figure 5). In fact, the chi-square indicator is considerably higher for S2 than for S5 (Figure 6), indicating that a larger number of the vowels produced by S2 fell out of the expected boundaries. A potential interpretation of these data is that S2 overly resorts to her L1 routines when performing the vowel reproduction task, so that her responses are poorly distributed over the entire acoustic space (see also Figure 2) and poorly correlated to the stimuli's properties. However, if doing so S2 appropriately selects the closest-to-stimulus L1 prototype, this strategy results in a minimization of the stimulus-response distance when compared to S5.

The latter example illustrates the fact that different indicators may reflect different aspects of the speakers' behavior in the reproduction tasks. Instead of selecting one indicator over the others, at this early stage of development we favor an assessment of phonetic compliance through a compound of complementary, well-selected indicators. In the remaining of this discussion, we draw on the first results achieved in study 1 to outline the requirements for refining the assessment of phonetic compliance within the limits of the data collection paradigm used here, to serve as guidelines for the implementation of study 2.

What is first needed is an assessment of the speakers' ability to approximate the targets' acoustic properties. Indicators from the regression analyses as well as distance indices are complementary in this matter, especially in the case of vowels since the distance index has the advantage of being computable in a multidimensional space. However, it is noteworthy that the computed Euclidean distance in the F1/F2/F3 space gives a similar weight in deviations along the three dimensions, i.e., F3 deviations are treated similarly as F1 deviations (on a mel scale). For a discussion of the appropriate way to address this issue, which in our view lies beyond the scope of the present study, see general discussion below.

Second, a valuable indicator should target the speakers' ability to deviate from their L1 routines in order to attain unusual targets. Such an indicator would help in interpreting results such as those collected from S2 and S5 in the vowel task (see above). It would also potentially document the kind of behavior displayed by S4 with the VOT set, who seems to attempt to move out of her presumed “territory” (short VOT, Figure 7), with little success (Figures 8, 9). However, in order to compute such an indicator, more data about the typical realizations of the speakers in L1 are needed, to be collected in study 2 (with regards to vowels, see below).

A third type of valuable indicators assesses the responses' overall distribution, given that stimuli properties are evenly distributed over a delimited space. Local Proportion, Global Proportion as well as the chi-square indicator were used in a first attempt to assess the surface density of the speakers' vowel productions in study 1 (Figure 6). However, the approach suffers some drawbacks, such as the projection onto a F1 by F2 plane, which was motivated by the short number of data points per rectangular zone, and the fact that, by definition density is computed based on the (binary) membership of one element to a set of elements, so that in this case a response just falling out of bounds was given the same status (“mismatch expectations”) as a response falling very far away from the target. By adding three more blocks to the first three blocks of study 1, the data collected in study 2 allowed us to better quantify the distribution of the responses over the F1∗F2∗F3 space, exploiting the variance observed in the six reproductions of the same target.

## Study 2

In order to further develop useful indicators to assess phonetic compliance, study 2 was carried out to gather complementary data on a subset of the stimuli and participants from study 1. Speakers who participated in study 2 were S3, S4, S6, and S10. They were selected because they exhibited a variety of behaviors based on the results of study 1. Among female speakers, S3 performed the best: her mean stimuli-responses distances in the F1/F2/F3 space were the lowest (Figure 5), her productions were well dispersed in the vowel space (Figure 2), and she exhibited the highest R-square value in the regression analysis of the VOT data. Conversely, S4 performed poorly in both tasks (note her tendency to centralize a majority of her vowel productions in Figure 2). Male speakers S6 and S10 performed equally good when considering the mean distances between target and reproductions in the F1/F2/F3 space (Figure 5), but the distribution of their productions over the vowel space was quite contrasted (Figure 2), which should be captured by refined indices of phonetic compliance.

Indeed, in addition to the first type of phonetic compliance indicators (indicators from the regression analyses; Euclidean distance index: “Index1” below), a second type of indicators were computed in study 2: “Index2,” which was designed to take into account the speakers' ability to move out of their L1-defined territory in achieving the reproduction task, and “Index3,” which assesses the distribution of the responses over the acoustic space in a refined way. The first type of indicators may be viewed as directly assessing the performances in phonetic compliance, whereas the second type are oriented toward the characterization of the underlying phonetic competence.

### Material and methods

#### Participants, stimuli and paradigm

The 4 speakers S3, S4, S6, and S10 participated in a single session comprising three parts. The first part consisted in a reading task of the 10 French oral vowels presented one at a time in ten consecutive blocks using the same grapheme-to-phoneme conversion rules as previously, for a total of 100 L1 vowels to be produced per speaker. The second and third parts were the same as in study 1, i.e., 10 training vowel reproductions and 3 blocks (Block 4, Block 5, Block 6) of 94 test vowel reproductions, with no feedback. The complementary 272 vowels were manually segmented and F1, F2, F3 formant frequencies were computed following the procedure described above.

#### Data processing

For each speaker, the centroids of the 10 L1 vowels were computed by averaging their formant properties over the 10 repetitions.

As to the vowels produced in the reproduction task, in addition to carrying out regression analyses similar to those reported for study 1, Index 1, Index 2, and Index 3 were computed based on the *total* dataset, i.e., 6 responses per stimulus for each speaker.

First, recall that in Index 1, the similarity between the stimuli to be reproduced by the speaker and the corresponding productions is estimated by the deviations of the productions from the targets. For a given speaker engaged in a reproduction task involving **S** vocoid stimuli, **P** productions of each stimulus, and provided that for each vowel, **I** (F_i_) formants are taken into account for each production, the average target/realization Euclidean distance is an indicator of the success of the speaker in the task. In Equation 8 (a generalization of Equation 7 above), Index 1 tends toward zero when the productions of the speaker tend, globally, toward the target in the vocalic space. In other words, Index 1 is zero when performance is maximal.

(8) $$\text{Index1}=\frac{\sum_{\text{s }=\;1}^{\text{S}}\sum_{\text{p }=\;1}^{\text{P}}{\left[\sum_{\text{i }=\;1}^{\text{I}}{\left({\text{Fi}}_{\text{ps}}\text{-}{\text{Fi}}_{\text{s}}\right)}^{2}\right]}^{\text{1/2}}}{\text{S}*\text{P}}$$

Index 2 is based upon the same principle, except that in this case, the *inverse* of the distance (−1/2 exponent) was taken into account, in order to obtain a number with variations positively correlated with performance in the task. Furthermore, in this case, the speaker was “calibrated” using his/her realizations of L1 vowels. Given that L1 has **V** vocalic phonemes and the speaker has realized **v** tokens of each, it is possible to identify zones of the vowel space corresponding to usual productions of the speakers, and zones where he/she is not used to produce vocalic sounds. The idea in Index 2 is to give higher reward to the success in imitating when imitation takes place in a region of the vocalic space the speakers do not spontaneously use in their usual practice of their L1. This is the reason for the weighting by the multiplicative term (Equation 9). It consists in the logarithm of the sum of all the distances between a given production and each vowel's cluster centroid: the multiplicative term tends toward zero when at least one distance production/centroid tends toward zero. Thus, for a given realization, the resulting product (of the logarithm by the inverse of the Euclidean production-target distance) is large if the production resembles the target and if it is produced in a zone far from the ones corresponding with the speaker's L1. Index 2 represents the average product over all the speaker's productions:

(9) $$\text{Index2}=\frac{\sum_{\text{s }=\;1}^{\text{S}}\sum_{\text{p }=\;1}^{\text{p}}\left\{\begin{array}{c} \prod_{\text{v }=\;1}^{\text{V}}\text{log}{\left[\sum_{\text{i }=\;1}^{\text{I}}{\left({\text{Fi}}_{\text{ps}}-\bar{{\text{Fi}}_{\text{v}}}\right)}^{2}\right]}^{\text{1/2}}{\left[\sum_{\text{i }=\;1}^{\text{I}}{\left({\text{Fi}}_{\text{ps}}\text{ - }{\text{Fi}}_{\text{s}}\right)}^{2}\right]}^{-\text{1/2}} \end{array}\right\}}{\text{S}*\text{P}}$$

In Index 3 (Equation 10, where “var” stands for “variance”), the similarity between targets and productions is no more the main point, and the approach is more statistical: it is based upon the analysis of variability in the reproduction task. When a speaker tries to attain a target, he/she produces realizations that fall around it in the reference space. If the speaker's compliance is high, his/her variability around the target in the reference space is random, and if no other source of variance is active, the variability is constant whatever the stimulus. On the other hand, if the speaker is strongly influenced by his/her L1, one can suppose that his/her variability will vary from one stimulus to another, depending on whether the stimulus is close or not to a region of the vowel space present in L1. Index 3 should therefore tend toward zero (all variances equal) for a speaker who performs well in the task.

(10) $$\text{Index3}={\text{var}}_{\text{s}}\left\{\sum_{\text{p }=\;1}^{\text{p}}{\text{var}}_{\text{p}}\left({\left[\sum_{\text{i }=\;1}^{\text{I}}{\left({\text{Fi}}_{\text{ps}}\text{ - }{\text{Fi}}_{\text{s}}\right)}^{2}\right]}^{1/2}\right)\right\}$$

Two advantages of Index 3 are noteworthy. First, Index 3 implements the assessment of the distribution of the responses over the acoustic space in a continuous rather than a discrete manner (as was the case when comparing the surface density of the responses with that of the stimuli in 34 mutually exclusive zones of the F1/F2 plane). Second, to a certain extent Index 3 circumvents the problem raised by the fact that, for some speakers more than for other speakers, the (anatomically or articulatory-induced) limits of their individual acoustic space diverge from the limits of the stimuli space. Since here the similarity between targets and responses is not taken into account, speakers who need to apply a large transformation to the targets' formant values in order to repeat them “faithfully” are not (as much) at a disadvantage when their performances in the reproduction task are assessed. Note, however, that it is still possible that some speakers would show a non-random dispersion pattern around specific targets that are beyond their reach.

### Results

**Figure 10:**
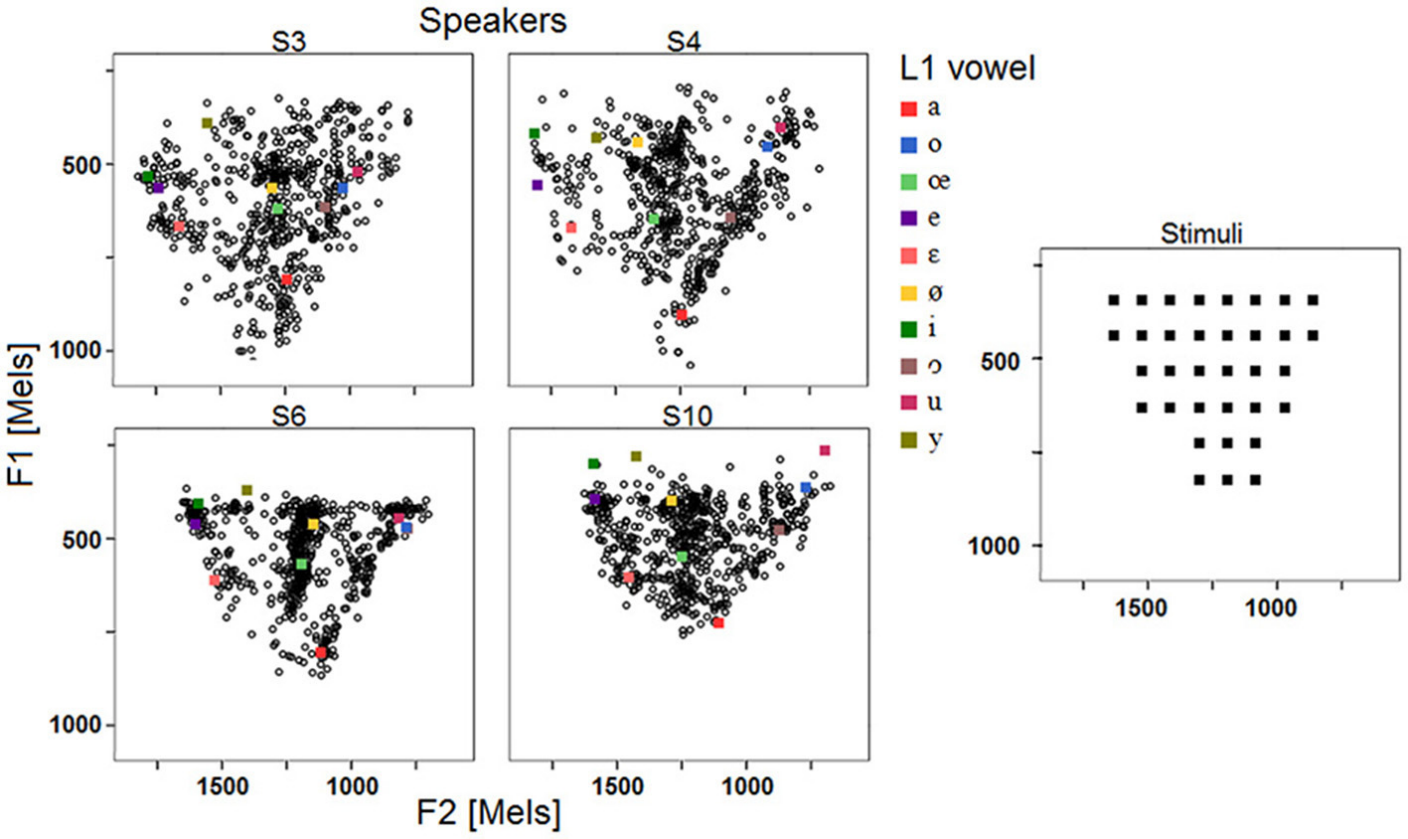
**F1 and F2 (mels) of the stimuli and response vowels, as well as the centroids of the L1 realizations, across the 4 speakers and the 6 blocks**.

The acoustic properties of the 564 vowels (94 stimuli ∗ 6 repetitions) produced by the four speakers, as well as the centroids of the L1 vowels, are plotted in the F1/F2 plane in Figure 10. As suggested above, the vowels produced by the speakers during the reproduction task generally fall within the boundaries of their native vocalic space. Clusters mostly arise in the region of the vocalic space devoted to central vowels.

**Figure 11:**
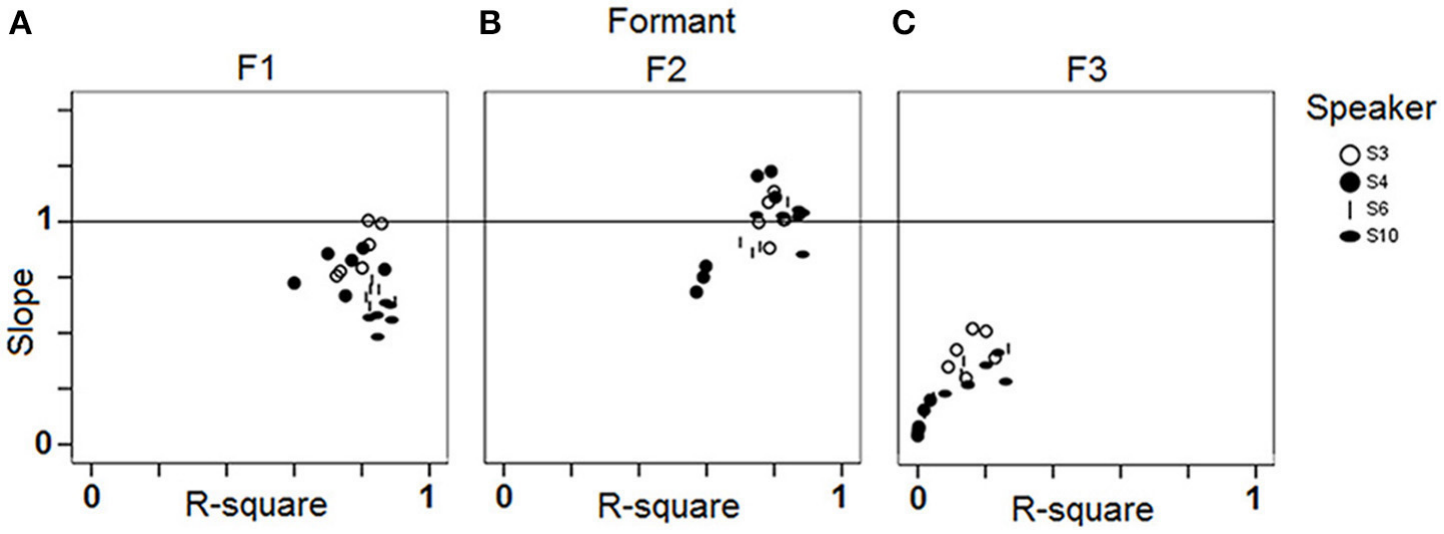
**Results of the single regression analyses carried out for each speaker and each block (study 2): slope of the linear regression line by R-square plots for (A) F1; (B) F2; (C) F3**.

Regression analyses were carried out, for each speaker, each block and each formant separately. Results are summarized in Figure 11, in the same form as in Figure 4. Group tendencies (one regression per formant, all speakers and all blocks pooled) are as follows. Overall the speakers perform best in correlating response F2 with stimulus F2 [R-square = 0.86; *F*_(1, 374)_ = 2273.17; *p* < 0.001; Slope = 0.99], they perform fairly regarding F1 [R-square = 0.8; *F*_(1, 374)_ = 1536.9; *p* < 0.001; Slope = 0.73] and poorly regarding F3 [R-square = 0.06; *F*_(1, 374)_ = 26.01; *p* < 0.001; Slope = 0.27]. As expected, adding more data from the four speakers under study did not alter much the group tendencies observed in study 1. Note that in Figure 11 the six data points related to a given speaker generally fall close to each other, suggesting intra-individual performance robustness, except in the case of F2 for S4, who performed better in this regard in the second data collection session.

Apart from S4, who can be considered as the least efficient in the reproduction task based on the regression results, the other three participants are not easily discriminable from the regression analyses. It may be considered either as a lack of power from the analysis to achieve the assigned goal, or as an advantage since the regression analysis is able to capture the good performances of the female speaker S3 despite the fact that a significant part of her productions' F1 and F2 fall quite above the limits of the stimuli space (Figure 10).

Complementary assessments of the speakers' performances are provided by Index 1, Index 2 and Index 3 (Table 3). We mainly comment here on the resulting ranking orders of the four speakers, since score values carry little meaning in themselves (except for Index 1, although an average Euclidean distance of 100–200 Hz in the F1/F2/F3 space remains difficult to interpret). Note that, at later stages of development of the assessment procedure, large empirical dataset could allow setting up potential reference score values.

**Table 3:** **Index 1, Index 2, Index 3 scores and ranking orders with respect to phonetic compliance (“1” is the most compliant) for the 4 speakers of study 2**.

	**Index 1**		**Index 2**		**Index 3**	
**Speaker**	**Score (Hz)**	**Rank**	**Score**	**Rank**	**Score**	**Rank**
S3	199.84	3	61.9	4	3872	3
S4	216.4	4	74.98	3	7475	4
S6	148.17	2	79.93	2	2457	2
S10	137.27	1	87.4	1	1552	1

Whether computed on six or on three data points, Index 1 results in a similar ranking order, the average distance between stimuli and responses in the F1∗F2∗F3 space being consistently larger for S3 and S4 than for S6 and S10 (Figure 5 and Table 3). Actually S6 and S10 are the highest ranked, and S3 and S4 are the lowest ranked for the three indices.

The indices mainly differ in how they rank S4. S4 performs particularly poorly in Index 3, meaning that the variance of the responses with regards to some of the targets is large, whereas it is considerably smaller around other targets, so that the variance of variances is particularly high. In contrast, S10 is the highest ranked based on Index 3, not so much so because on average his variances are low, but because they are all very similar, so that the variance of variances is low.

Only Index 2 ranks S4 over S3, indicating that when performing the task S4 resorts less than S3 to her L1-typical realizations as assessed by the L1 vowel centroids displayed in Figure 10. If one compares the values obtained by S3 and S4 regarding the three indices, a possible interpretation is that S4 takes more risks when she attempts at faithfully repeating the stimuli, so that she is more prone to move out of her L1 routines (and is rewarded through Index 2). However, these out-of-habits excursions increase the variance of variances measured by Index 3. By contrast, S3 may be considered as a female speaker who is overall more in control of her productions (Index 3), and more efficient (Index 1), but who accomplishes the task by keeping closer to her phonetic routines in L1 (Index 2).

### Discussion

Data collected from study 2, pooled with those from study 1, allowed us to carry out four different ways of assessing the performances of four speakers in the vowel reproduction task. The four procedures are different and complementary, both in terms of outcomes (i.e., in terms of the representations of the individual performances they result in), and in terms of underlying vision of phonetic compliance.

The advantage of the regression analysis over a distance-based approach is that it provides an assessment of the correlations between stimuli and responses, which by definition can be high even if, in terms of formant frequencies, responses considerably diverge from the stimuli to be repeated (see the case of S3 above). However, when the responses do diverge considerably from the targets, it remains unclear whether the task (“repeat the stimuli as *faithfully* as possible”) should be considered as successful. Probably the answer to this question lies in the perceptual domain, which is out of the scope of this paper.

The three indices developed here each condense a large amount of information into a single value. Index 1 simply consists in a global assessment of the distance between the stimuli and the responses in the F1∗F2∗F3 space. It mainly focuses on the *performance* component of phonetic compliance, but is not totally exempt from assumptions, particularly regarding the relative weights of the three formants in the formula. Considering now the underlying *competence* that drives the speakers' performances, one has to go further than this “phonetic,” even “acoustic,” perspective on the data, and take into account the fact that each speaker is equipped with an L1-dependent phonological system structuring his/her speech production and speech perception experiences. The phonological system results in some regions of the vocalic space presumably acting as perceptual magnets (Kuhl, 1991) and aggregating preferred phonetic realizations. Index 2 and Index 3 integrate this constitutive aspect of the cognitive processing of human language.

Index 2 uses independently-assessed direct information about each individual's native phonological system to assess his/her performances in the vowel reproduction task, since the response-to-stimuli acoustic distances are weighted according to their originality with respect to the speaker's preferred phonetic realizations. Although the first results are promising, this approach could probably benefit from some mathematical refinement, e.g., with regards to the variance of L1 realizations around the centroids and to the specific weight to assign to the multiplicative term in Equation 9. The latter should also undergo some normalization process, i.e., the reward given by the multiplicative term should be expressed, not in absolute terms, but as a proportion of the maximal reward that could be offered to any particular speaker given the specifics of his/her L1 categories, so that comparisons between speakers would be more meaningful.

Index 3 approaches phonetic compliance from the perspective of phonetic control. Good performers are those speakers who succeed in keeping at bay the disturbances (such as the perceptual magnet effects) that are related to their phonological system structuring patterns. Recall however that these patterns are not directly built into Index 3. Rather, the index uses responses dispersion in order to assess how the speakers are in control of their productions. Speakers who succeed in maintaining constant the dispersion of their responses around the corresponding stimulus *whatever the stimulus* are considered to perform the best. In this approach, phonetic variability is not viewed as disturbing noise, but as a possible source of information. A low variance of variances is taken as an indicator of controlled phonetic behavior.

In summary, in study 2 we have collected complementary data necessary to further develop a variety of quantification techniques aimed at assessing how the selected speakers process unfamiliar sounds, not only in terms of performances, but also in terms of the underlying (cognitive, phonological and phonetic) competence driving these performances.

## General discussion

The aim of this paper was to contribute to control for part of the inter-individual variability that is frequently reported in experimental studies dealing with nonnative speech sounds learning. We have assumed that this variability is at least to some degree due to *phonetic compliance*, i.e., to the intrinsic speaker-specific ability to appropriately mobilize perception and production processes in order to produce speech sounds which are unusual in L1. Although individual phonetic abilities are usually considered as playing an influential role in L2 acquisition (e.g., Jilka, 2009), we found no previous attempt in the literature to investigate phonetic compliance as itself, independently of the speakers' proficiency in a specific L2, and based on direct measurements on speech production data.

In terms of an underlying conceptual model, we have defined our work as an attempt toward isolating a newly identified, specific, C component from the classical X = T + E model. Even if C is linked with the inter-individual variability, we claimed that it has erroneously been viewed as random and partly confused with E. On the contrary, we have assumed that C is systematic and part of the determinants of T (which expresses the result of the engagement of a subject in a specific experimental paradigm). In other words, the C component has been assumed here to represent a systematic source of variance which is inherently linked to the subject's behavior, and influences the measurement of his performances in the phonetic task he/she's engaged in. We claim that an appropriate assessment of the C component would allow for a better estimate of the effects of the independent variables under investigation in experiments on L2 sound learning.

### Contributions of the proof-of-concept study

In this paper, we have presented a two-fold proof-of-concept study which constitutes the first stage of a research program aimed at the development of phonetic compliance assessment tools. The present study was designed to test the feasibility of assessing phonetic compliance by collecting empirical data in a controlled, laboratory, environment, on an occasional sample of participants and by carrying out a first data analysis using descriptive statistics (study 1), then by drawing from this initial analysis a first set of indicators that may be useful to appropriately assess, and further refine the concept of, phonetic compliance (study 2).

In study 1, we have explored the intrinsic speaker-dependent variability exhibited in two reproduction tasks on different speech materials, using a variety of statistical analyses. Since subjects had to reproduce oral vowels as well as aspirated plosive consonants, they had, on the one hand, to attain new, not L1-typical, targets in an acoustic space they already mastered, and on the other hand, to reorganize their timing patterns between already mastered laryngeal and supra-laryngeal gestures. As intended, this pilot data collection paradigm elicited significant inter-speaker intra-task differences, as well as interesting intra-speaker inter-tasks discrepancies, so that functional individual profiles may be hypothesized. These profiles may be derived from the contrasting performances in the vowel vs. the VOT reproduction tasks (e.g., S8), as well as from the comparison of the performances, within the vowel reproduction task, using different quantification methods (e.g., S2 vs. S5).

Study 2 was carried out to collect the complementary data that were necessary in order to further develop three customized indices that may prove valuable for the assessment of phonetic compliance. Index 1 consists in a global assessment of the overall distance between the stimuli and the responses in the formants space. As such, Index 1 represents an appropriate way of assessing the performance component of phonetic compliance, in purely acoustic terms. Index 2 and Index 3, on the contrary, take into account the fact that each speaker is equipped with an L1-dependent phonological system structuring his/her speech production and speech perception experiences. By addressing the way in which speakers are able to cope with the dynamics of their own system, Index 2 and Index 3 are oriented toward the competence component of the speakers' phonetic compliance.

All 3 indices may have potential to discriminate between subjects differing in phonetic compliance. They are nevertheless quite different from one another, both in terms of underlying cognitive hypotheses (strong in Index 2 and Index 3, absent in Index 1) and of workload requirements (Index 2 requires prior calibrating of the subject, Index 1 and Index 3 do not). When implemented here (on a limited sample of four native French speakers), they resulted in partially different outcomes, in that the four speakers were not ranked in exactly the same order by the different indices.

**Figure 12:**
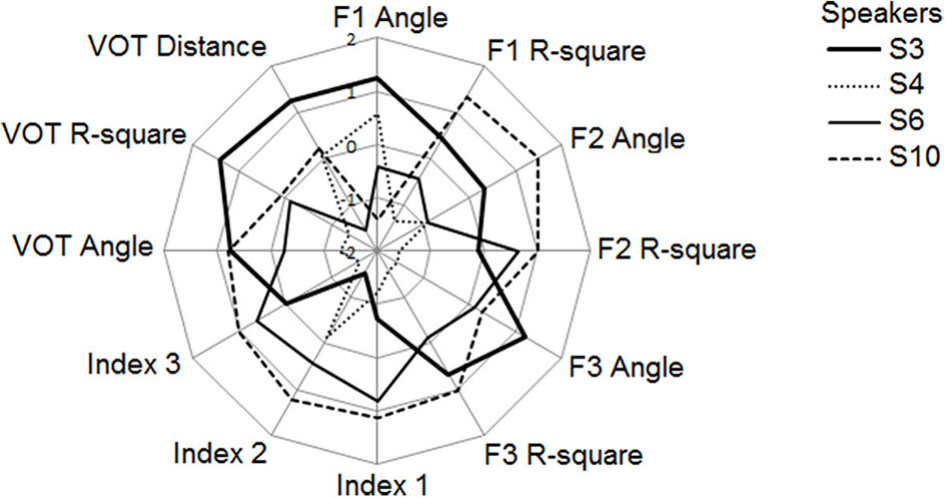
**Summary of the performances of the 4 speakers (study 2) along 12 indicators (values expressed in z-scores).** See text for details.

Given the complementarity of the different indicators developed in this paper to assess the speakers' performances in the reproduction tasks, we claim that it would be inappropriate at this stage to select one indicator over the others. Rather, we propose a first, exploratory operationalization of the notion of phonetic compliance as a compound of the most meaningful indicators drawn from the two studies reported here. Figure 12 illustrates this approach for S3, S4, S6, and S10 using a radar chart. Rather than preferring one mathematical technique over the others, the analysis summarized in Figure 12 suggests an alternative view: the one of a multi-componential approach that would favor an assessment of phonetic compliance through a compound of complementary indicators, such as those taken into account in study 1 and in study 2.

In total, 12 indicators were used to draw the chart, i.e., for the vowel task (6 blocks): 6 parameters related to the regression analyses (slopes of the regression line, coefficients of determination for F1, F2, F3) as well as the three global indices Index1, Index2, and Index 3; for the VOT task (5 blocks), a parameter related to the slope of the regression line and the coefficient of determination from the ResponseVOT by StimulusVOT regression, as well as the mean distance between StimulusVOT and ResponseVOT. Individual z-scores were computed for each of these indicators to allow for inter-indicators comparisons. Since the idea is to express performance as an increasing value along each axis, the slopes of the regression lines have been expressed in degrees of the related slope angles, then the absolute difference between those angles and 45° have been computed, so that the slope-related indicators (“F_i_ Angle” in Figure 12) actually represent how the speakers approximate a slope of 1 in the corresponding regression analyses, therefore treating the magnitude of overshoot in the same way as the magnitude of undershoot. Also note that the slope-related indicators, as well as Index 1, Index 3 and the mean distance between stimulus VOT and response VOT, are plotted in reversed scale in Figure 12, so that the scores on the radar are positively correlated with good performances for all indicators.

Figure 12 provides a useful visualization of inter-individual differences in the various aspects composing phonetic compliance as expressed in this pilot data collection paradigm. Figure 12 shows that S4 is the less compliant speaker, with below average performances for all indicators but two. S3 and S10 are the most compliant, S3 performing better in the VOT task whereas S10 performs better in the vowel task.

### Limitations and perspectives

By definition, a proof-of-concept study such as the one reported in the present paper does not seek generalizability. Rather, it should be used to formulate the guidelines for the following stages of development of the device which feasibility has been established. Thus, based on a critical analysis of the major limitations of the methods used in the present study, we outline in this section the improvements, modifications and extensions that are necessary to adapt the methodology developed here in order to conduct a large scale study on phonetic compliance in native French speakers, then we discuss the additional tasks and procedures that would be required in order to build a reliable and valid test of phonetic compliance applicable to speakers from virtually any linguistic background.

First, the limited group of participants in our study was not expected to approximate the distribution of phonetic compliance in the reference population. A large scale study is necessary to gather data from a much larger pool of (say, 100) native French speakers. The participants' history and everyday experience with any foreign language should be well documented, as well as their level of competence in oral comprehension and production (ideally, through a variety of indicators such as academic grades, self-assessment measures and scores obtained by external examinations using standardized scales). An assessment of **“**degree of foreign accent” could also be made by native speakers based on a sample of spontaneous production.

This extended complementary dataset would allow comparing the indicators resulting from the phonetic compliance assessment procedure with each participant's level of mastery of L2 phonetics and phonology, notwithstanding years of education and everyday use of a particular foreign language, thus allowing to test for criterion validity. Indeed, in the absence of any other test of phonetic compliance for comparing the results of the procedure described here, convergent validity tests are impossible to carry out at the moment. Besides, the reliability of the assessment procedure could be established by adapting the current method in two ways, i.e., by increasing the number of blocks within a data collection session, which would give more generalizability to the accumulated data, and by holding successive data collection sessions so as to investigate test-retest reliability.

Second, the vowel stimuli set should be amended. The present stimuli set was built to encompass the whole range of F1∗F2∗F3 combinations that is available to any adult human speaker who is comfortable with the fixed F0 contour (from 110 to 90 Hz). However, speakers whose vocal tract significantly differ from the norm derived from “typical” male adult speakers (around 17.5 cm length), particularly female speakers with shorter vocal tracts, but also male speakers with longer than average vocal tracts, may be considered at a disadvantage in the present study because the boundaries of their individual acoustic space notably differ from those of the stimuli space. Actually, only a few speakers' typical vowel space are likely to closely match the boundaries of any fixed stimuli space. Since the application of a post-task normalization procedure would run the risk of hindering what we precisely intent to measure here, an alternative experimental method should be considered, in which the stimuli set is adapted to each particular speaker (in terms of F0 range and boundaries of the F1∗F2∗F3 space), based on preliminary data collected from a short vowel production task in L1. For instance, all vowel stimuli spaces might comprise an equal amount of stimuli that are evenly distributed over the acoustic space, but the steps between formant values would be individually set. It should be noted that this speaker-specific adaptation of the stimuli vowel space is only intended to ensure that no target is out-of-bounds for any speaker engaged in the reproduction task. There is no straightforward way to “even things” further, i.e., to guarantee that the task is equally “easy,” or “difficult,” for all the speakers. Some speakers more than others may have to modify their articulatory routines, i.e., to apply larger transformations to their usual phonetic variants, in order to be successful in the reproduction of the stimuli, which matches the situation they face when learning new phonetic variants in L2. However, it is our belief that the human speech production and perception systems are indeed highly flexible and fit to the task, as exemplified by the efficiency of communication between children and adults in L1, or between a male student and a female teacher in L2 classrooms.

Third, the metrics developed in this proof-of-concept study deserve mathematical refinement. As a priority, the target-production Euclidean distances in the three-formant space (upon which Index 1 and Index 2 are based) would have to be related to the size of each individual's acoustic space following the modification proposed above, e.g., using the speaker-specific between-formant step values as a reference. In addition, Index 1 and Index 2 could be computed using alternative measures of target-production distances in which F1, F2, and F3 are given unequal weights. Indeed, results presented here indicate that in the reproductions, F3 values are generally less consistent from one block to another than F1 and F2 values, and that in any block Response F3 values are better predicted by Response F1 and Response F2 values then by Stimulus F3 values. Based on the empirically-grounded analysis offered by Schwartz and colleagues concerning the relative weights of F1, F2, and F3 (in the perceptual domain: Schwartz et al., 1997), a good starting point could be to attribute them a weight of respectively 1, 1/3, and 1/6 when computing Index 1 and Index 2. Elaborating on Index 2, not only the centroids of the L1 vowels, but also the variances around these centroids should be part of the equation, and the introduction of reference phonetic categories from any foreign language known by the participant (whose weight could be lighter than L1 categories, and adjusted to the degree of mastery of L2 phonetics and phonology) should be considered. The resulting rewarding term should be normalized for the number of preexisting phonetic categories as well as for their configuration. Also, the relative weight of the two multiplicative terms, the categories-related term and the target-reproduction distances term, would deserve further deliberation.

It should be clear at this point that the proposals for further refining the metrics proposed in the present paper do not go as far as specifying the appropriate values that would be needed for a direct implementation. Actually, we believe that a substantial part of the data analysis of the larger scale study mentioned above should be dedicated to study how the resulting rankings of participants in terms of phonetic compliance would be sensitive to the variation of the various weighting parameters, in order to make well-informed choices about the values to finally opt for. Note that here, as in the presentation of the results from Study 2, we primarily aim at ranking speakers relatively to each other (in their attempt to reproduce a common acoustic model), rather than assessing their performances along an independent scale. Indeed, it may be impractical, or even theoretically absurd, to state *a priori* the expected mean, spread, maximum and minimum of the performances in the phonetic compliance task (e.g., Is perfect imitation likely to be in the reach of any human speaker? How to determine a minimum/chance level? Etc.). Instead, large scale studies on a refined version of the paradigm developed here should allow determining the range of scores that are typically attained in a given population, allowing for further comparisons between an individual participant's score and the group's performances.

Finally, moving to the additional requirements needed to build a reliable and valid test of phonetic compliance applicable to virtually any human speaker, we focus here on three major extensions, involving respectively additional stimuli in the reproduction tasks, supplementary groups of speakers, and extra tasks aimed at consolidating construct validity.

First, an extension to other stimuli is in order, so that the sounds to be reproduced better encompass the range of human speech sounds. Suprasegmentals should be included starting with syllables of varying (static and dynamic) tones, as well as source-related phonetic variants such as creaky and breathy vowels. Concerning consonants, VOT is only one in many phenomena deserving investigation (and not critically so for speakers whose L1 already contrasts short lag vs. long lag voiceless stops). A comprehensive test should involve the reproduction of consonants of varying places, manners of articulation and airstream mechanisms (ejectives, implosives, clicks), that could be for instance embedded in a_a pseudo-words. The ability to cope with short range dynamics should also be tested, e.g., using diphtongs and V-to-V coarticulation patterns.

On the negative side, a major rise in the amount of stimuli would undoubtedly result in a significant increase in the number of data collection sessions (particularly at the earlier stages of development when internal consistency and test-retest reliability should be established) and in the amount of data to be analyzed, and would potentially end up in a plethora of candidates as indicators of phonetic compliance. However, these extensions may be necessary since from a theoretical point of view phonetic compliance should be considered as the ability to produce faithfully human speech sounds in all their diversity, and from a methodological point of view the most compliant individuals may be considered as those speakers who perform best with a variety of stimuli. In other words, diversifying the sound sets to be reproduced would ensure content validity. Besides, many standardized aptitude tests in their extended version do actually include a number of “subtests” which scores may be combined in several ways to compute indices of sub-skills that are considered to be part of the general aptitude under study. In the case of speech sounds, different subscores could be combined to gather information on an individual's ability to cope with static vs. dynamic sound properties, vocalic vs. consonantal variants, laryngeal vs. supralaryngeal mechanisms, etc. Increasing the number and types of stimuli to be reproduced could also be the only solution if one seeks to build a test which would allow comparing individuals from (very) different linguistic background in terms of phonetic compliance. Depending on the participants' L1 (and proficiency in other foreign languages), they may have an advantage or not in reproducing some, but not all of, the sounds sets mentioned above.

Obviously, a large amount of empirical, methodological and even theoretical work is called for in order to establish the appropriate procedures (i) to gather the data, analyze them, and develop the appropriate resulting indicators for each subtest; (ii) to elaborate single and combined scores from the different subsets that would finally condense in a few numerical values a valid and reliable measure of phonetic compliance. Empirical work would necessarily involve gathering data from pools of speakers of various linguistic background, ideally including speakers who are bilinguals, monolinguals and diversely proficient in a number of foreign languages, as well as languages that are typologically different in terms of sound systems (tone languages, languages of different vowel and consonant inventory sizes, languages of different syllable structure and degree of complexity, etc.). In order to consolidate the construct validity of the future test of phonetic compliance, we would recommend to include additional tasks to be performed by the participants. Convergent validity would require the most and least compliant speakers to perform respectively among the best and the poorest in tasks that are related to L2 sound learning, such as perceptual training tasks (e.g., Wayland and Guion, 2004; Iverson et al., 2005; Lambacher et al., 2005; Wang, 2008), or tasks of reproduction of short real words from an unknown human language (such as Hindi, following Jilka, 2009). Divergent validity could be established by showing that scores resulting from the phonetic compliance test are unrelated to scores achieved in other, standardized, (sub-)tests for foreign vocabulary or syntax learning (e.g., the “Paired Associates” subtest of the Modern Language Aptitude Test (Carroll and Sapon, 2002), in which participants must quickly learn a set of vocabulary words from another language and memorize their meanings in L1).

## Conclusion

As a conclusion, the present proof-of-concept study was carried out as the initial stage of a research program aimed at developing the appropriate tools to assess phonetic compliance, an individual ability which, in our view, plays an influential role in L2 sound learning but so far has not received the attention it deserves. The tremendous amount of work that is likely required to develop an appropriate test of phonetic compliance is justified in our opinion by the methodological and theoretical issues it would help to address. From a methodological point of view, we claim that experimental studies in SLA would benefit from a test which allows to control for the participants' phonetic compliance, either in the selection procedure or in data analysis, and would thus contribute to a better understanding of the performances of language learners with respect to nonnative sounds. From a theoretical point of view, once phonetic compliance can be appropriately and independently assessed, one will be able to investigate its relationships with other individual characteristics such as foreign language aptitude, individual strategies in L2 learning, or even propensity to phonetic convergence in L1, as well as cognitive processes like phonological working memory and selective attention.

### Conflict of interest statement

The authors declare that the research was conducted in the absence of any commercial or financial relationships that could be construed as a potential conflict of interest.

## Footnotes

^1^ interface provided at http://www.asel.udel.edu/speech/tutorials/synthesis/expert.html

^2^ Some of the stimuli in the left upper half of the vowel stimuli space (see Figure 1) have also been excluded because F2 and F3 values would be too close (less than 250 mels), which is also quite rare in the world's languages, and is absolutely prohibitive using Klatt's synthesizer, since it results in an unwanted energy peak in the region where two close poles significantly reinforce each other.
